# The Pre-Transplant Non-HLA Antibody Burden Associates With the Development of Histology of Antibody-Mediated Rejection After Kidney Transplantation

**DOI:** 10.3389/fimmu.2022.809059

**Published:** 2022-02-16

**Authors:** Aleksandar Senev, Bryan Ray, Evelyne Lerut, Jayasree Hariharan, Christine Heylen, Dirk Kuypers, Ben Sprangers, Marie-Paule Emonds, Maarten Naesens

**Affiliations:** ^1^ Department of Microbiology, Immunology and Transplantation, KU Leuven, Leuven, Belgium; ^2^ Histocompatibility and Immunogenetics Laboratory, Belgian Red Cross—Flanders, Mechelen, Belgium; ^3^ Immucor Inc., Norcross, GA, United States; ^4^ Department of Imaging and Pathology, University Hospitals Leuven, Leuven, Belgium; ^5^ Department of Nephrology and Renal Transplantation, University Hospitals Leuven, Leuven, Belgium

**Keywords:** non-HLA antibodies, autoimmunity, kidney transplantation, graft outcome, rejection, graft failure, histology, transplant glomerulopathy

## Abstract

**Background:**

Many kidney allografts fail due to the occurrence of antibody-mediated rejection (ABMR), related to donor-specific anti-HLA antibodies (HLA-DSA). However, the histology of ABMR can also be observed in patients without HLA-DSA. While some non-HLA antibodies have been related to the histology of ABMR, it is not well known to what extent they contribute to kidney allograft injury. Here we aimed to investigate the role of 82 different non-HLA antibodies in the occurrence of histology of ABMR after kidney transplantation.

**Methods:**

We included all patients who underwent kidney transplantation between 2004-2013 in a single center and had biobanked serum. Pre- and post-transplant sera (n=2870) were retrospectively tested for the presence of 82 different non-HLA antibodies using a prototype bead assay on Luminex (*Immucor, Inc*). A ratio was calculated between the measured MFI value and the cut-off MFI defined by the vendor for each non-HLA target.

**Results:**

874 patients had available pretransplant sera and were included in this analysis. Of them, 133 (15.2%) received a repeat kidney allograft, and 100 (11.4%) had pretransplant HLA-DSA. In total, 204 (23.3%) patients developed histology of ABMR after kidney transplantation. In 79 patients (38.7%) the histology of ABMR was explained by pretransplant or *de novo* HLA-DSA. The multivariable Cox analysis revealed that only the broadly non-HLA sensitized (number of positive non-HLA antibodies) patients and those with the highest total strength of the non-HLA antibodies (total ratios of the positive non-HLA antibodies) were independently associated with increased rates of histology of ABMR after transplantation. Additionally, independent associations were found for antibodies against TUBB (HR=2.40; 95% CI 1.37 – 4.21, p=0.002), Collagen III (HR=1.67; 95% CI 1.08 – 2.58, p=0.02), VCL (HR=2.04; 95% CI 1.12 – 3.71, p=0.02) and STAT6 (HR=1.47; 95% CI 1.01 – 2.15, p=0.04). The overall posttransplant non-HLA autoreactivity was not associated with increased rates of ABMRh.

**Conclusions:**

This study shows that patients highly and broadly sensitized against non-HLA targets are associated with an increased risk of ABMR histology after kidney transplantations in the absence of HLA-DSA. Also, some pretransplant non‐HLA autoantibodies are individually associated with increased rates of ABMR histology. However, whether these associations are clinically relevant and represent causality, warrants further studies.

## Introduction

Many kidney allografts fail due to the occurrence of antibody-mediated rejection (ABMR) and donor-specific anti-HLA antibodies (HLA-DSA) (1). But the histology of ABMR, the first two Banff criteria for ABMR, and transplant glomerulopathy, the hallmark lesion of chronic form of ABMR, can be observed in the absence of circulating HLA-DSA (2–6). Consequently, the role of antibody responses against non-HLA targets in mediating ABMR has become a central focus in kidney transplantation in the latest years (7). While the number of publications linking non-HLA antibodies to the development of ABMR histology (ABMR_h_) is growing, it is still unclear how these non-HLA antibodies contribute to kidney allograft damage and graft failure. Currently, there is inadequate consensus for routine testing of any non-HLA antibody for diagnosing ABMR (8–12), although there is growing literature on the role of anti-angiotensin receptor II type 1 antibodies as risk factor for ABMR and graft failure and for potential future clinical implementation (13, 14).

In the last decade, several autoantibodies against non-HLA targets have been implicated in kidney allograft rejection, such as angiotensin II type 1 receptor (AT1R), endothelin-1 type A receptor (ETAR), agrin, myosin, perlecan, vimentin and tubulin (8, 15). The number of published articles on this topic and antibodies against novel non-HLA targets, like most recently ARHGDIB, is continuously increasing (16–18). The most extensively studied target is AT1R (19), and several studies have linked anti-AT1R antibodies to poor allograft outcomes in kidney transplantation (20). However, controversy exists over its clinical relevance as its presence is not always linked to kidney graft rejection or graft failure and final conclusions are hampered by the case-control study design and selection bias in most studies (21, 22). Moreover, recently it has become apparent that interplay between HLA-DSA antibody and other non-HLA antibody responses likely exist. It has been shown that in patients with HLA-DSA, the concomitant presence of antibodies, anti-AT1R or anti-ARHGDIB, portends inferior kidney graft outcome suggesting a synergistic effect of these antibodies on allograft injury (18, 20). While certain studies have indicated that the anti-AT1R antibodies can induce allograft injury independently of HLA-DSA, this was not replicated in all studies (20, 23). Therefore, the real effect solely of anti-AT1R or other antibodies on kidney allograft damage, in the absence of HLA-DSA, remains largely unknown.

Additional complexity in understanding the clinical relevance of the non-HLA immunity in solid-organ transplantation was raised by studies showing that non-HLA genetic mismatches between the donor and recipient and the alloimmune responses against these targets are a significant predictor of kidney allograft outcome (24–26). Reindl-Schwaighofer et al. found that non-HLA genetic mismatches in the immune-accessible transmembrane proteins can develop donor-specific alloantibodies and lead to graft failure (25). Similarly, Steers et al. have shown that the genomic mismatches at *LIMS1* locus may lead to allosensitization in hypoxia-induced conditions and kidney graft rejection (26). These findings question the relevance of the non-HLA autoimmunity, and suggest that the clinically relevant non-HLA antibodies in kidney transplantation may be alloimmune.

Here, we sought to assess the clinical relevance of non-HLA antibodies in a large cohort of well-characterized kidney transplant patients and examine their relation to histopathology findings and long-term graft survival. To avoid selection bias, we tested all available pre- and post-transplant serum samples of our single-center cohort for the presence of a large panel of non-HLA antibodies.

## Materials and Methods

### Study Design and Study Population

**Figure 1:**
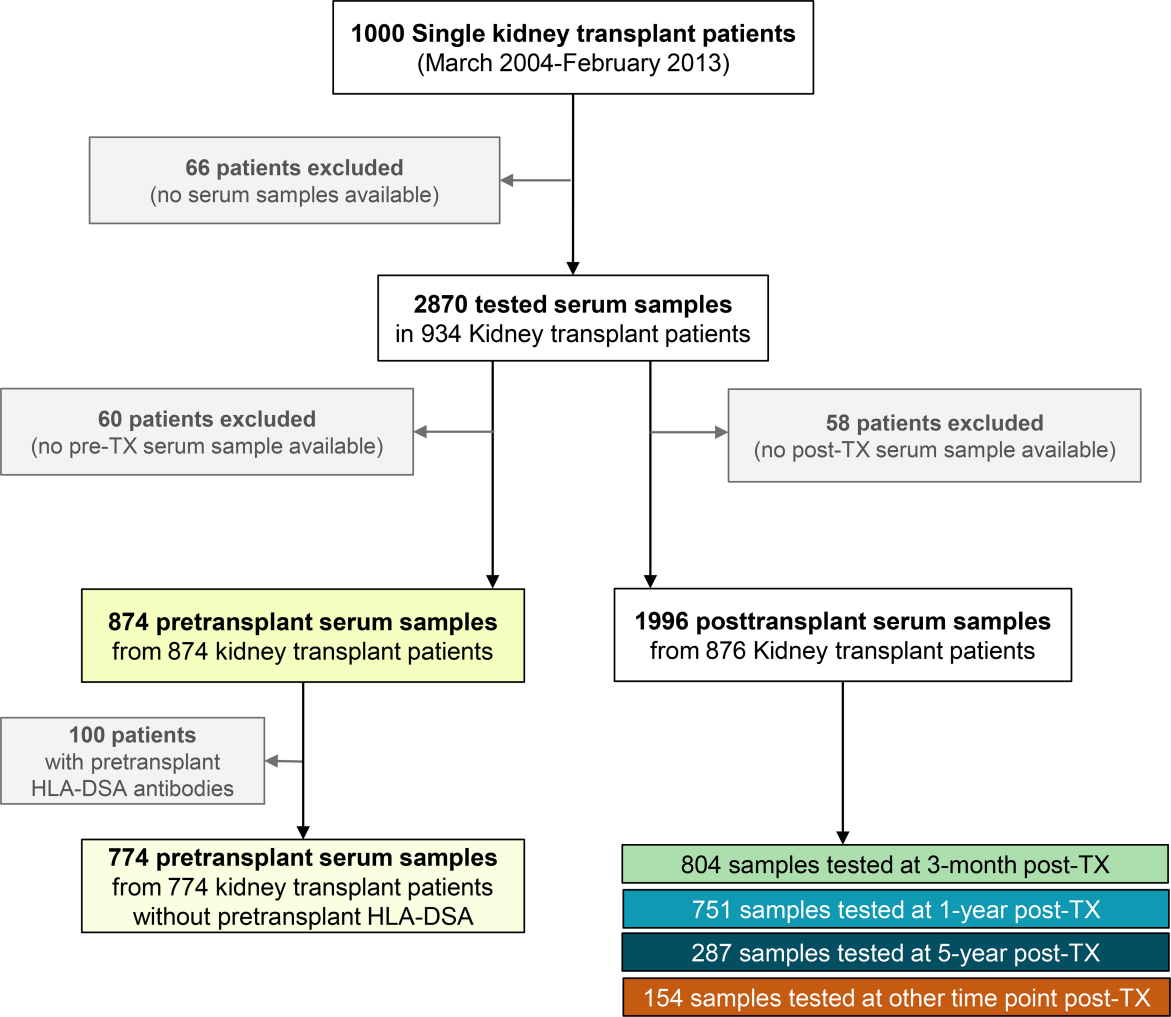
Flow chart for patient enrollment and study design. Of the 934 kidney transplant patients included in this study, 874 patients had a pretransplant serum sample and 876 patients had at least one post-transplant serum sample available for non-HLA testing.

We conducted a retrospective observational cohort study of all adult patients who underwent single kidney transplantation at the University Hospitals Leuven between March 1, 2004, and Feb 6, 2013 (N=1000), and who had available pre- or post-transplant biobanked serum samples (N=934) (**Figure 1**). All transplantations were performed with compatible T and B-cell complement-dependent cytotoxicity tests and with ABO blood groups compatibility. Clinical data were collected prospectively and systematically stored in electronic formats. In the vast majority of patients, tacrolimus, mycophenolic acid, and corticosteroids were administered as a basic immunosuppressive regimen, with extra induction treatment in higher-risk individuals. No desensitization therapies for HLA antibodies were used. The Ethics Committee of the University Hospitals Leuven approved this retrospective study (S60562).

### High-Resolution HLA Genotyping of the Cohort

Using Next-Generation Sequencing (NGS) technology, the recipients (N=926) and donors (N=926) in this cohort with available DNA samples were retrospectively genotyped at second field high-resolution HLA level for 11 HLA loci: HLA-A, -B, -C, -DRB_1_, -DRB_3_, -DRB_4_, -DRB_5_, -DQA_1_, -DQB_1_, -DPA_1_, and -DPB_1_. We used two HLA genotyping approaches: the MIA FORA NGS FLEX 11 HLA Typing Kit (Immucor Inc., Norcross, GA, USA) on the MiSeq sequencing instrument (Illumina Inc., San Diego, CA 92121, USA) and the HLA genotyping approach of Histogenetics (Ossining, NY, USA) on the HiSeq sequencing system (Illumina Inc.). We have described this retyping strategy in detail previously (27). The remaining donors without sufficient DNA samples for complete genotyping of all 11 HLA loci, were retrospectively genotyped at second field high-resolution HLA level only for the locus or loci needed for assessment of the presence of HLA-DSA in the corresponding recipients using the MIA FORA NGS FLEX 11 HLA Typing Kit.

### Anti-HLA Antibody Detection and Identification of Circulating Anti-HLA Antibodies

For all recipients, the LIFECODES LifeScreen Deluxe kit (Immucor) was used to screen the recipients for anti-HLA antibodies both before and after transplantation. Antibody identification was done with the LIFECODES Single Antigen Bead kits (Immucor) on the day of transplantation, 3 months post-transplant, and then yearly after transplantation when there was a positive or suspected false-negative screening result (for instance in patients with historic, pretransplant or previously positive sample, retransplant patients, patients with rejection episodes, samples with high background etc.). Consequently, at least one sample of each patient was tasted using the Single Antigen Bead kit. To avoid the prozone effect, we systematically utilized ethylenediaminetetraacetic acid (EDTA). We considered background-corrected median fluorescence intensity values ≥ 500 to indicate a possible presence of antibodies with donor-specificity (HLA-DSA). More details on the final assignment of HLA-DSA in this cohort were described recently (28).

### Detection of Non-HLA Antibodies

The pre- and post-transplant serum samples were retrospectively tested for the presence of 82 different non-HLA antibodies using a prototype bead assay under development by Immucor. All the tests were performed by Immucor according to the manufacturer’s instructions and the raw data were collected using a Luminex 200 instrument. Briefly, 10 µL serum is mixed with 40 µL bead mix and incubated for 30 minutes are room temperature. Unbound serum is then removed and phycoerythrin conjugated anti-human IgG is added for detection. All the tests were run in one large batch in one laboratory performed by one experienced technologist using the same lot to minimize the intra- and inter-laboratory variability. A ratio was calculated between the measured MFI value and the cut-off MFI defined by the vendor for each non-HLA target. To establish the cutoff, 100 sera from non-transfused males were used. Because some of the sera were suspected to have autoantibody, the highest 15% MFI for each antigen were removed and the averages for the remaining values were calculated. The cutoff for each antigen was independently set at 3-times the average for the given antigen. The 82 non-HLA antigens included in the panel, along with the MFI cutoffs, are listed in **Table S1**. Antibodies were assigned as positive for a given target if the calculated ratio was ≥1. For each patient, the broadness of non-HLA sensitization was calculated by summing the number of targets with ratio ≥1. The strength of non-HLA antibody sensitization (total positive ratio) was calculated by summarizing all ratios with a value ≥1.

### Kidney Allograft Biopsies, Histologic Grading and Treatment of Rejection Episodes

We included all protocol and indication renal allograft biopsies performed during post-transplant follow-up in this cohort until September 1, 2019. Indication biopsies were performed at time of graft dysfunction (eGFR, proteinuria and their evolution), as evaluated by the attending physician. Protocol biopsies were defined as prescheduled biopsies at time of stable graft function and performed at 3, 12, 24, 36 or 48 and 60 months after transplant (27). All biopsies were reviewed and retrospectively rescored by one pathologist (EL). The individual histological lesion scores were semi-quantitatively assessed according to the most recent Banff 2019 consensus (29). Borderline changes for acute TCMR were diagnosed as foci of tubulitis (t>0) with minor interstitial inflammation (i1) or moderate-severe interstitial inflammation (i2 or i3) with mild (t1) tubulitis, also in concordance with the Banff 2019 consensus. We did not separately consider chronic active TCMR. Typically, acute TCMR was treated with high-dose steroids, both on indication and protocol biopsies, with subsequent second-line therapy using ATG in steroid-resistant cases. Borderline changes were often treated when diagnosed at the time of graft dysfunction (indication biopsies), but not in protocol biopsies in the context of stable graft function. For the biopsies that met the first two Banff 2019 criteria for ABMR_h_, we used the term histologic picture of ABMR (ABMR_h_), as described previously (3). Patients with ABMRh who were HLA-DSA negative but were C4d positive were classified as “full” ABMR according to Banff 2019 criteria. Only a limited number of cases with ABMR received specific therapy, as reported previously, due to the retrospective rescoring of the biopsies and by the lack of access to efficacious therapies.

### Statistical Analysis

Descriptive statistics are reported using frequencies and percentages for categorical variables and using means and standard deviations or medians and interquartile ranges for continuous variables. The Wilcoxon test was used for the comparison of medians between the different groups. We used univariable and multivariable Poisson regression analysis to estimate the relationships between different patient factors and the presence of pretransplant non-HLA antibodies. Cox proportional hazards models were applied to quantify the hazard ratios for the occurrence of the individual histologic lesions and phenotypes according to the strength and the broadness of pretransplant non-HLA antibodies. In these survival analyses, the patients were censored at the time of the last biopsy. To address confounding factors, we adjusted all multivariable models for HLA-A, -B, -DR, -DQ antigen mismatches, repeat transplantation, deceased donation, recipient sex, the recipient and donor age, and induction therapy. The pretransplant non-HLA antibodies were included linearly or as quartiles in the Cox models. Sensitivity analyses were performed in the pretransplant HLA-DSA negative patients; for these survival analyses, the patients were additionally censored at the time of *de novo* HLA-DSA occurrence. The univariable Cox regression analysis was also used to investigate possible individual non-HLA antibody predictors for ABMR_h_ or graft failure. All non-HLA antibodies with a p-value of ≤ 0.10 in the univariable analysis were included in a multivariable Cox model. The stepwise selection method was used to select the final independent predictors. Spearman’s correlation coefficient was used to measure the strength of the relationship between the pretransplant non-HLA antibodies and pretransplant gamma globulin values. We defined graft failure as the loss of kidney transplant function (return to dialysis or re-transplantation). In case of death of the recipient with a functioning graft, graft failure was censored at the time of death. We administratively censored patients who did not experience graft failure and were still in follow-up on September 1, 2019, at the time of data extraction. All p values less than 0.05 were considered to be statistically significant. Statistical analyses were performed using SAS (v9.4; SAS Institute Cary, NC, USA) and graphs were produced using GraphPad Prism software (v9.4; GraphPad Software, San Diego, California, USA).

## Results

### Study Population and Non-HLA Antibody Testing

934 single kidney transplantations were included in this retrospective cohort study. The main demographics and clinical characteristics of this study population are shown in **Table 1**. Most of the patients received deceased kidney allograft 877 (93.9%), and 141 (15.1%) patients received a repeat kidney allograft. 247 (26.4%) patients had pretransplant anti-HLA antibodies, of which 108 (43.7%) had specificity against the HLA antigens of the transplanted graft (HLA-DSA). The median follow‐up time was 8.1 (IQR 5.3-10.7) years.

**Table 1:** Baseline characteristics and follow-up of the study population (n=934).

Cohort characteristics	Total (n=934)
***Recipient demographics***	
Sex (male), n (%)	571 (61.1)
Age (years), mean ± SD	53.5 ± 13.2
Repeat transplantation, n (%)	141 (15.1)
Body mass index (kg/m^2^), mean ± SD	25.4 ± 4.5
Caucasian ethnicity, n (%)	918 (98.3)
Cause of end stage renal disease	
Immune-mediated kidney diseases (IgA nephropathy, FSGS etc.), n (%)	251 (26.9)
Cystic kidney disease	176 (18.8)
Tubulointerstitial disease (TID)	147 (15.7)
Chronic kidney disease (CKD)	93 (10.0)
Diabetic nephropathy	86 (9.2)
Vascular disease	64 (6.9)
Hereditary kidney disease	32 (3.4)
Other disease	85 (9.1)
***Donor demographics***	
Sex (male), n (%)	498 (53.3)
Age (years), mean ± SD	47.6 ± 14.9
Living donor, n (%)	57 (6.1)
Donation after brain death, n (%)	724 (77.5)
Donation after cardiac death, n (%)	153 (16.4)
Cold ischaemia time (h), mean ± SD	14.2 ± 5.7
***Transplant characteristics***	
HLA-ABDRDQ antigen mismatches (0–8), mean ± SD	3.4 ± 1.6
Pretransplant total gamma globulins (g/L), mean ± SD	11.0 ± 3.1
Pretransplant anti-HLA antibodies, n (%)	247 (26.4)
Pretransplant donor-specific HLA antibodies, n (%)	108 (11.6)
MFI pretransplant donor-specific HLA antibodies, median (IQR)	3125 (1345-6468)
Induction therapy, n (%)	389 (41.6)
Immunosuppression regimen: TAC-MPA-CS, n (%)	800 (85.7)
*De novo* donor-specific HLA antibodies, n (%)	46 (4.9)
*MFI de novo donor-specific HLA antibodies, median (IQR)*	2326 (1363-6449)
Median follow-up time post-transplant (years, IQR)	8.1 (5.3 – 10.7)

TAC, tacrolimus; MPA, mycophenolic acid; CS, corticosteroids; SD, standard deviation; IQR, interquartile range; MFI, median fluorescence intensity; FSGS, focal segmental glomerulosclerosis;

In total, 2870 serum samples (874 pretransplant; 1996 posttransplant in 876 transplants) of the study cohort were available and tested for the presence of 82 different non-HLA antibodies. Of the total 934 patients included, 60 patients did not have pre-transplant serum and 58 patients did not have post-transplant serum available for non-HLA antibody testing.

### Pretransplant Non-HLA Antibodies and Post-Transplant Histology

**Figure 2:**
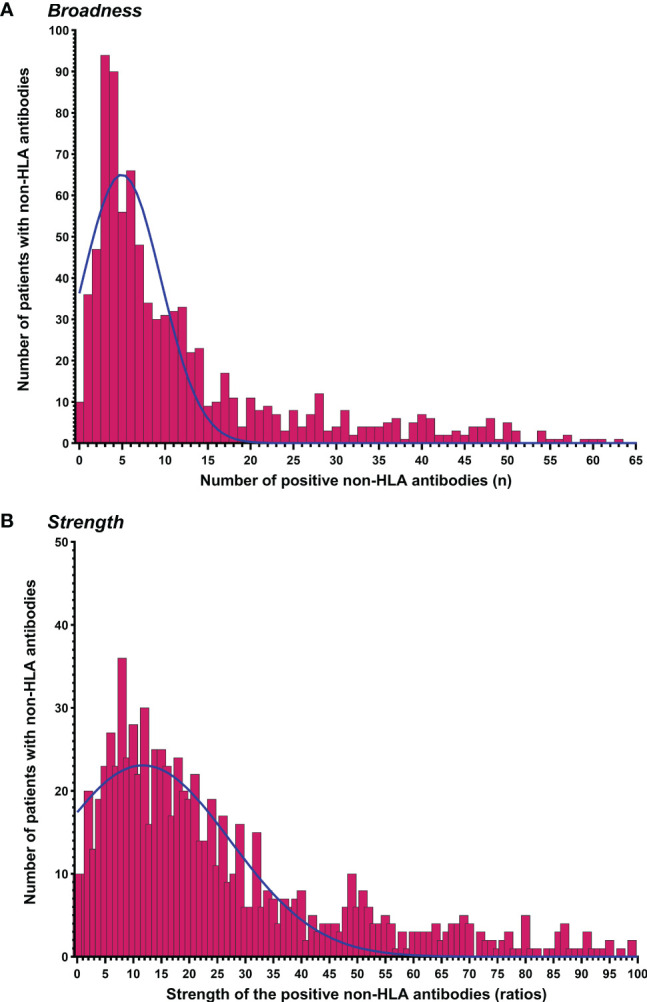
Distribution of patients by the number of positive pretransplant non-HLA antibodies. **(A)** The broadness of non-HLA antibody profile (number of positive non-HLA antibodies per patient). **(B)** The strength of non-HLA antibody profile (total ratios of the positive non-HLA antibodies per patient). 46 patients with ratios between 101 and 224 were not plotted on the graph.

**Figure 2** depicts the distribution of the total number of positive non-HLA antibodies per patient, which represents the broadness of non-HLA immunity, and of the total ratios of the positive non-HLA antibodies representing the strength of the non-HLA antibodies. Interestingly, of 874 patients with available pretransplant samples, 863 (98.7%) patients showed at least one target with raw MFI above the threshold MFI per antigen predetermined by the vendor. The distribution of the raw MFI values and the ratios above the cut-off for each non-HLA antigen are shown in **Figure S1**. In Pearson correlation analysis, some antibodies were positively correlated with each other; however, the ratios of the non-HLA antibodies were not correlated with the total ratio of pre-transplant HLA-DSA antibodies (**Figure S2**).

Next, we performed univariable and multivariable Poisson regression analyses for estimating possible independent predictors for the broadness and the strength of the non-HLA antibody profile (**Table 2**). The multivariable analysis showed only an increased Rate Ratio (RR) of hemodialysis with the broadness (RR=1.18, 95%CI 1.03 – 1.37, p=0.02) and the strength (RR=1.21, 95%CI 1.05 – 1.41, p=0.01) of the non-HLA antibody response. Repeat transplantation showed decreased RR with the broadness and the strength of the non-HLA antibody profile. Time on dialysis (RR=1.10 per 1000 days, 95%CI 1.03 – 1.18, p=0.006) and patient age (RR=1.06 per 10 years, 95%CI 1.01 – 1.11, p=0.02) showed independent association only with the strength of the total non-HLA antibody response.

**Table 2:** Multivariable Poisson regression analysis for estimating the relationships between different patient factors and the presence of pretransplant non-HLA antibodies (N=874).

Predictors	RR	95% CI	p-value
**Univariable analysis**			
***Risk rates for the broadness of the non-HLA antibodies***			
Time on dialysis (per 1000 days)	1.05	0.99 – 1.13	0.11
Type of dialysis (hemodialysis vs. other)	1.18	1.02 – 1.36	0.03
Repeat transplantation	0.69	0.58 – 0.82	<0.0001
Recipient age (10 years)	1.06	1.01 – 1.11	0.02
Recipient sex (male)	1.16	1.03 – 1.31	0.02
***Risk rates for the strength of the non-HLA antibodies***			
Time on dialysis (per 1000 days)	1.08	1.01 – 1.16	0.03
Type of dialysis (hemodialysis vs. other)	1.22	1.05 – 1.41	0.009
Repeat transplantation	0.74	0.63 – 0.89	0.0009
Recipient age (10 years)	1.06	1.01 – 1.11	0.01
Recipient sex (male)	1.12	0.99 – 1.28	0.07
**Multivariable analysis**			
***Risk rates for the broadness of the non-HLA antibodies***			
Type of dialysis (hemodialysis)	1.18	1.03 – 1.37	0.02
Repeated transplantation	0.71	0.59 – 0.84	<0.0001
Recipient age (10 years)	1.04	0.99 – 1.09	0.13
Recipient sex (male)	1.12	0.99 – 1.27	0.08
***Risk rates for the strength of the non-HLA antibodies***			
Type of dialysis (hemodialysis)	1.21	1.05 – 1.41	0.01
Time on dialysis (per 1000 days)	1.10	1.03 – 1.18	0.006
Repeated transplantation	0.73	0.62 – 0.88	0.0006
Recipient age (10 years)	1.06	1.01 – 1.11	0.02

RR, Rate Ratio.

Next, we investigated the associations between the broadness (**Table S2**) and the strength (**Table S3**) of the pretransplant non-HLA antibody sensitization and the post-transplant development of histopathologic lesions and phenotypes. The multivariable analysis showed only independent associations of the broadness (HR=1.14 per positivity of 10 antibodies; 95%CI 1.03-1.27; p=0.01) and the strength (HR=1.05 per positivity of 10 ratios for antibodies; 95%CI 1.01-1.09; p=0.009) of the pretransplant non-HLA antibody profile and development of ABMR_h_ (n=204) after kidney transplantation. No associations were found with other Banff phenotypes and the individual Banff lesions (**Tables S2**, **S3**).

### Pretransplant Non-HLA Antibodies and Posttransplant Allograft Histology in Absence of HLA-DSA

Of the total 204 (23.3%) patients who developed ABMR_h_ after kidney transplantation (with a median of 84 (7 - 365) days post-transplant), in 79 patients (38.2%) ABMR_h_ was explained by the presence of pretransplant or *de novo* HLA-DSA. To investigate the possible contribution only of non-HLA immunity to the occurrence of histopathological lesions and phenotypes, we restricted the Cox analyses to patients without pretransplant HLA-DSA and additionally censored them at the time of *de novo* HLA-DSA occurrence. Of 774 pretransplant HLA-DSA negative kidney transplant recipients with available biopsy follow-up, 125 (15.2%) developed ABMR_h_; 29 of them had ABMR in the absence of HLA-DSA but were C4d positive (ABMR according to Banff 2019); and 218 (28.2%) developed TCMR.

The Cox analysis showed independent associations between the broadness of the pretransplant non-HLA antibodies and the risk of developing ABMR Banff 2019 in absence of HLA-DSA (HR=1.30 per 10 antibodies; 95%CI 1.04-1.63; p=0.02), ABMR_h_ (HR=1.21 per 10 antibodies; 95%CI 1.08-1.36; p=0.001) and microvascular inflammation Banff score ≥2 (HR=1.13 per 10 antibodies; 95%CI 1.01-1.26; p=0.04) (**Table 3**). Of the analyses with the individual Banff lesions, only glomerulitis Banff score > 1 associated independently with increased broadness of non-HLA antibodies (HR=1.19 per 10 antibodies; 95%CI 1.02-1.13; p=0.03).

**Table 3:** Univariable and multivariable Cox proportional hazards analysis for histologic lesions and phenotypes, according to the broadness of pretransplant non-HLA antibodies in the absence of HLA-DSA (N=774).

Predictor: Broadness of pretransplant non-HLA antibodies (per 10 antibodies increment)						
Histologic outcome of the model	Univariable analysis			Multivariable analysis		
	HR	95%CI	p-value	HR	95%CI	p-value
**Histologic phenotypes** (events)						
ABMR_h_ (125)	1.23	1.10 – 1.37	0.0004	1.21	1.08 – 1.36	0.001
ABMR 2019 (29)	1.29	1.03 – 1.61	0.03	1.30	1.04 – 1.63	0.02
TCMR (218)	1.01	0.91 – 1.12	0.87	1.00	0.89 – 1.11	0.92
TCMR and borderline (330)	1.04	0.95 – 1.13	0.39	1.02	0.94 – 1.11	0.64
Microvascular inflammation score ≥2 (148)	1.15	1.03 – 1.28	0.01	1.13	1.01 – 1.26	0.04
IFTA score 2 (425)	1.02	0.94 – 1.11	0.64	1.02	0.94 – 1.11	0.64
**Individual lesions** (events)						
Glomerulitis (g) score> 0 (203)	1.09	0.99 – 1.21	0.08	1.07	0.96 – 1.18	0.23
Glomerulitis (g) score> 1 (70)	1.24	1.07 – 1.44	0.005	1.19	1.02 – 1.39	0.03
Peritubular capillaritis (ptc) score> 0 (214)	1.03	0.93 – 1.14	0.61	1.02	0.92 – 1.13	0.73
Peritubular capillaritis (ptc) score> 1 (95)	1.09	0.94 – 1.26	0.25	1.08	0.93 – 1.25	0.31
Endarteritis (v) score> 0 (139)	1.07	0.94 – 1.21	0.31	1.04	0.92 – 1.18	0.54
Endarteritis (v) score> 1 (20)	1.15	0.85 – 1.54	0.37	1.11	0.82 – 1.50	0.49
c4d score> 0 (262)	1.04	0.95 – 1.14	0.46	1.03	0.94 – 1.13	0.51
c4d score> 1 (50)	1.19	0.99 – 1.43	0.06	1.19	0.98 – 1.43	0.07
Interstitial inflammation (i) score> 0 (317)	1.02	0.94 – 1.11	0.67	1.00	0.92 – 1.10	0.96
Interstitial inflammation (i) score> 1 (184)	1.04	0.93 – 1.16	0.54	1.02	0.91 – 1.14	0.75
Tubulitis (t) score> 0 (531)	1.01	0.94 – 1.08	0.86	0.98	0.91 – 1.05	0.56
Tubulitis (t) score> 1 (262)	1.05	0.96 – 1.15	0.27	1.02	0.93 – 1.12	0.72
Chronic allograft glomerulopathy (cg) score>0 (70)	0.88	0.70 – 1.10	0.24	0.83	0.66 – 1.06	0.14
Chronic allograft glomerulopathy (cg) score>1 (25)	0.89	0.62 – 1.28	0.53	0.86	0.59 – 1.27	0.46
Arteriolar hyalinosis (ah) score> 1 (253)	1.02	0.92 – 1.12	0.74	1.00	0.91 – 1.11	0.94
Interstitial fibrosis (ci) score> 1 (361)	1.01	0.93 – 1.10	0.76	1.01	0.93 – 1.10	0.77
Tubular atrophy (ct) score> 1 (296)	0.99	0.91 – 1.09	0.89	1.00	0.91 – 1.10	0.94
Vascular intimal thickening (cv) score> 1 (350)	1.02	0.94 – 1.11	0.69	1.00	0.92 – 1.09	0.93
Mesangial matrix expansion (mm) score> 0 (183)	1.07	0.96 – 1.20	0.21	1.03	0.92 – 1.16	0.58

All multivariable Cox models were adjusted for anti-HLA antibodies, HLA-A, -B-DR, -DQ antigen mismatches, repeated transplantation, deceased donation, recipient sex, recipient and donor age and induction therapy.

C4d, complement 4d deposition.

Subsequent Cox analysis with the strength of the non-HLA antibodies, confirmed the independent association between the pretransplant non-HLA antibodies and the risk rates of developing ABMR 2019 in absence of HLA-DSA (HR=1.09 per 10; 95%CI 1.02-1.18; p=0.04) and ABMR_h_ (HR=1.06 per 10; 95%CI 1.02-1.11; p=0.005) after kidney transplantation (**Table S4**).

**Figure 3:**
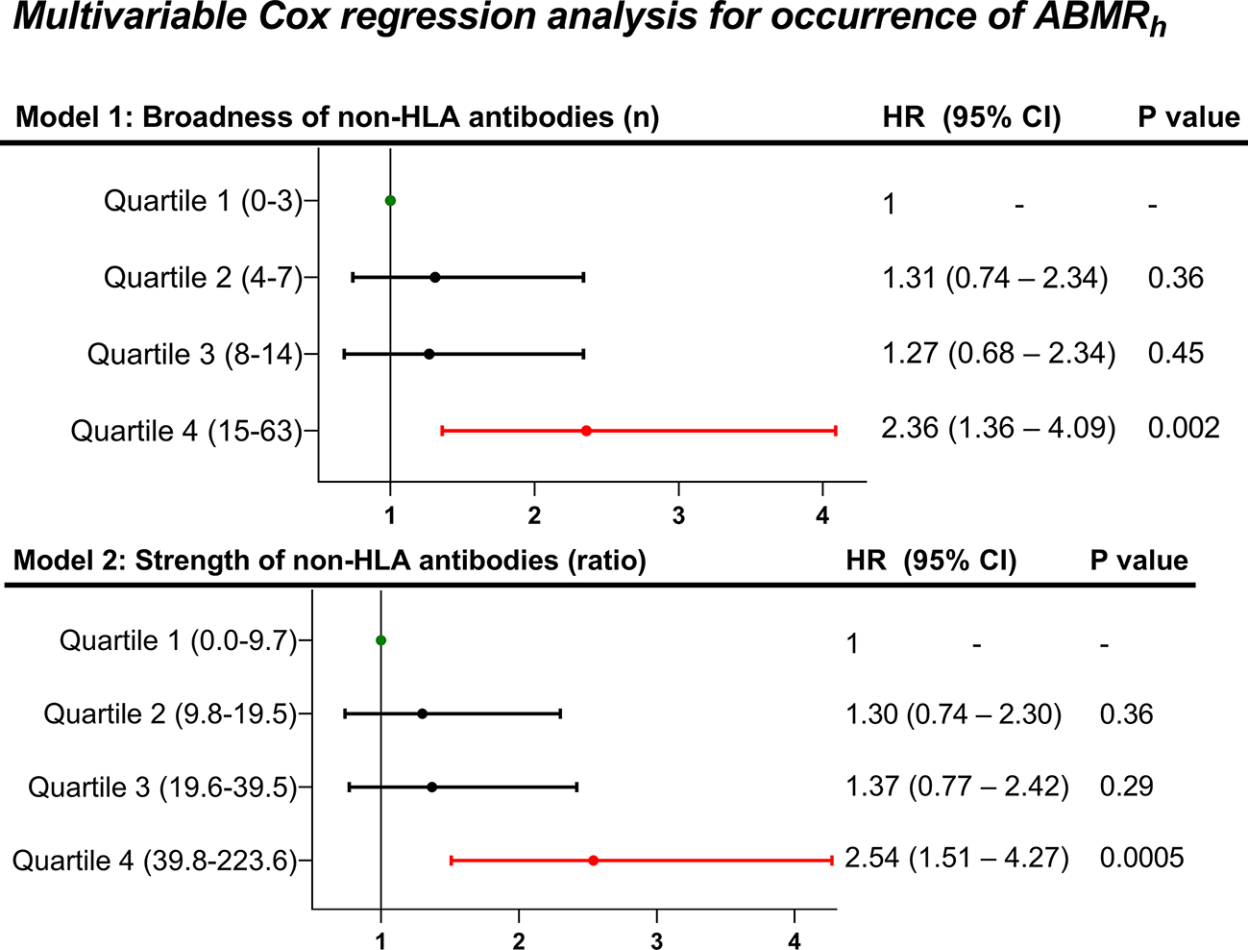
Multivariable Cox regression analysis of the occurrence of ABMR_h_ in the absence of HLA-DSA, according to different quartiles of the broadness and strength of pretransplant non-HLA antibodies (N=774). Each multivariable Cox model was adjusted for the presence of anti-HLA antibodies, HLA-A, -B, -DR, -DQ antigen mismatches, repeated transplantation, deceased donation, recipient sex, recipient and donor age, and induction therapy.

**Figure 4:**
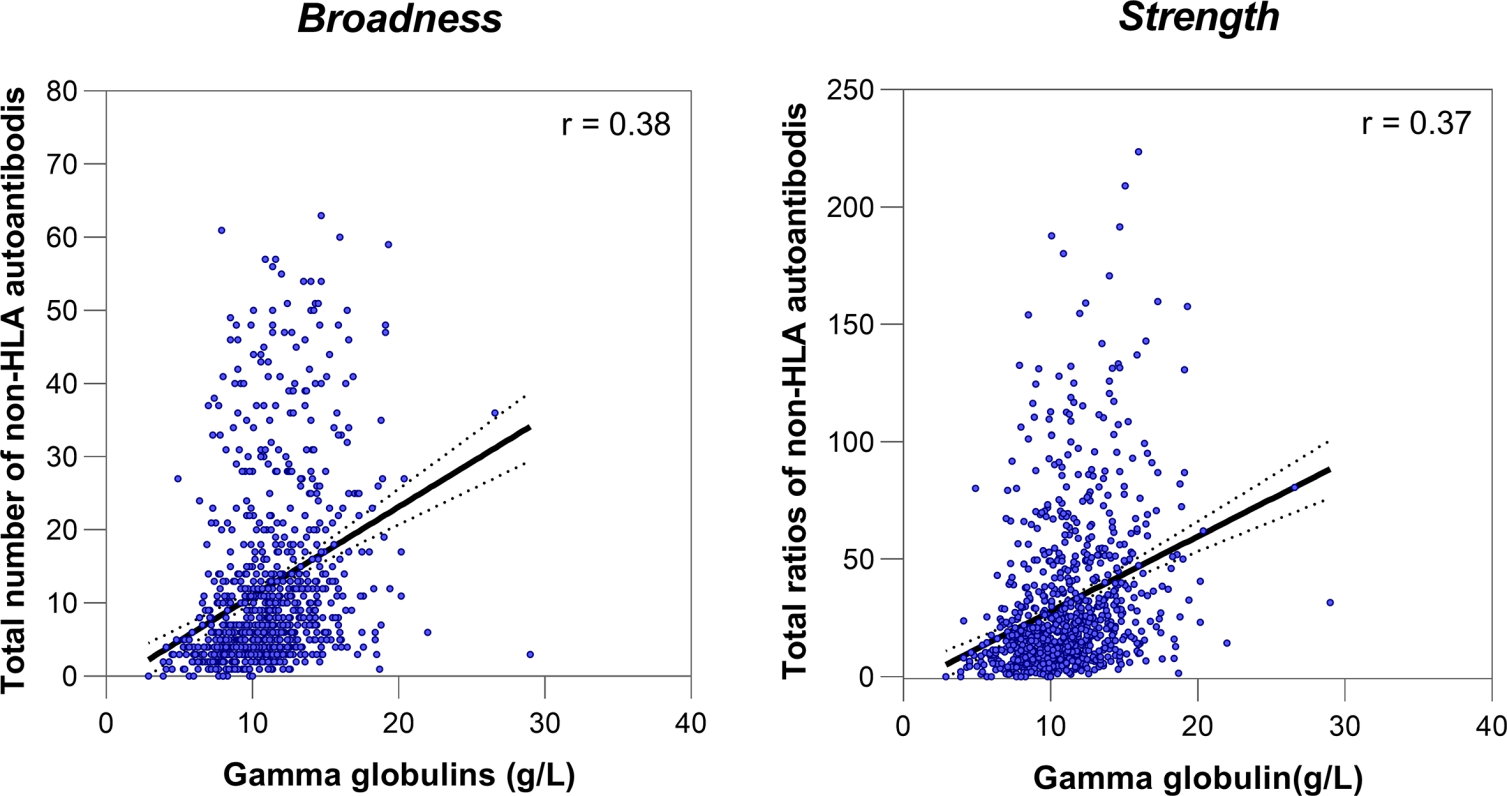
Correlations between the pretransplant non-HLA antibodies and total serum gamma globulins.

Next, we investigated how the different levels of non-HLA antibody profiles associate with the risk of developing ABMR_h_. For this, we stratified our cohort in quartiles (Q) according to the different degrees of the broadness (Q1 = 0-3, Q2 = 4-7, Q3 = 8-14 and Q4 = 15-63) and the strength (Q1 = 0.0 – 9.7, Q2 = 9.8 – 19.5, Q3 = 19.6 – 39.5 and Q4 = 39.8 – 223.6) of the non-HLA antibodies (**Table S5**). Compared to Q1 patients, this analysis showed that only the patients with the highest level of non-HLA antibodies, Q4 for broadness (HR=2.36; 95%CI 1.36-4.09; p=0.002) and Q4 for strength (HR=2.54; 95%CI 1.51-4.27; p=0.0005), associated independently with an increased rate for developing ABMR_h_ (**Figure 3**). Although total serum gamma globulins, measured by electrophoresis in g/L, showed a correlation with the broadness (r=0.38) and the strength (0.37) of non-HLA antibodies (**Figure 4**), the pretransplant total gamma globulins level did not associate with the development of ABMR_h_ in the univariable analysis (HR=1.03 per 1, 95% CI 0.97 - 1.08, p=0.38) (**Table S5**).

**Figure 5:**
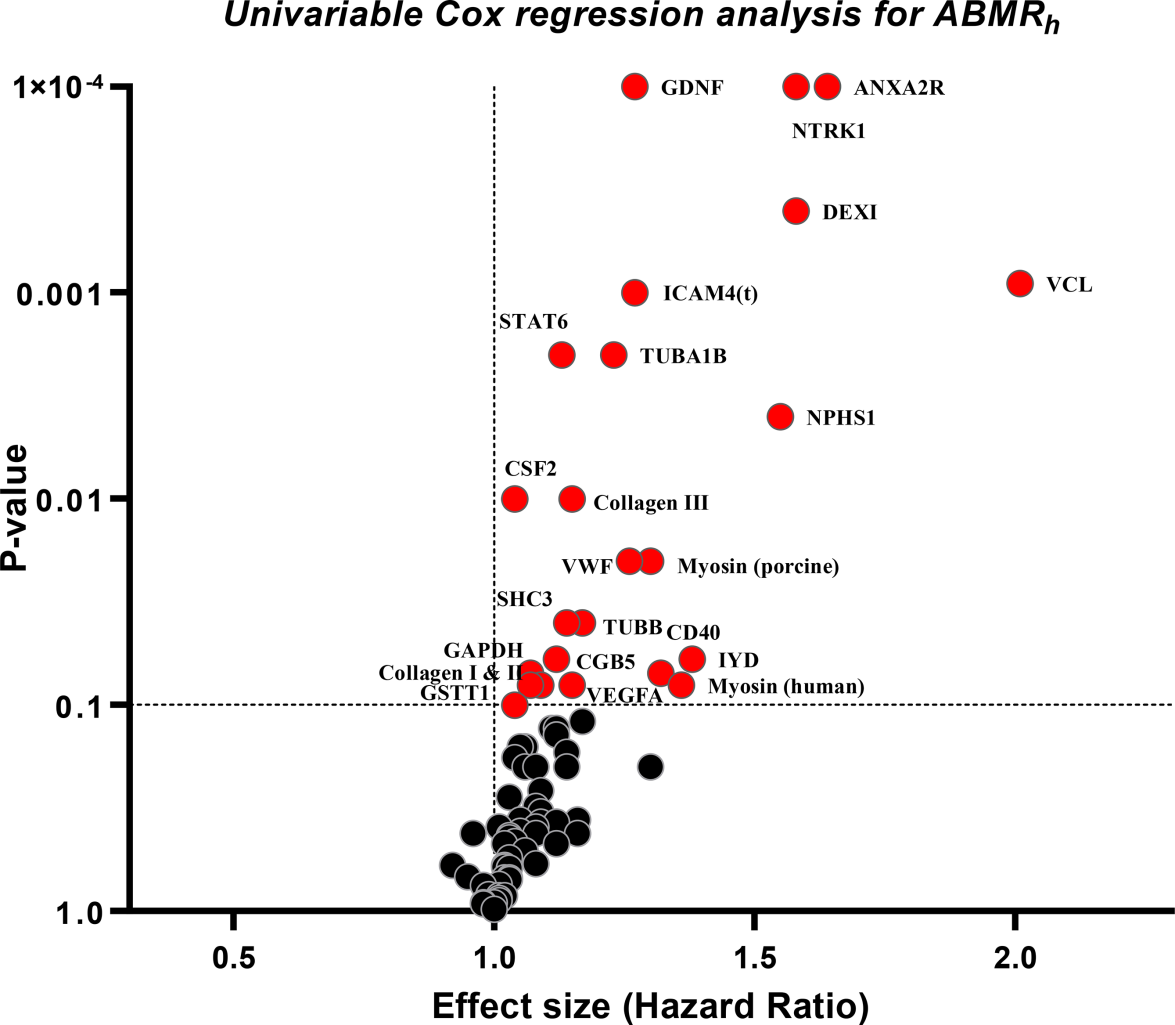
Volcano plots for associations between the individual pretransplant non-HLA antibodies and the occurrence of ABMR_h_ in the absence of HLA-DSA (N=774). The plot depicts the p-values and the Hazard ratios of the univariable Cox regression analysis for antibodies against 78 different targets. For three non-HLA targets, IL-21, Actin and FN1, no estimations were obtained from the Cox analysis due to the low number of positive patients. In this analysis patients were censored at the time of the last performed biopsy or at *de novo* HLA-DSA occurrence.

Further, we investigated the associations between the individual pretransplant non-HLA antibodies and the rates of developing ABMR_h_ after kidney transplantation (**Figure 5** and **Table S6**). The univariable analysis suggested antibodies against 24 non‐HLA antigenic targets to be associated with the ABMR_h_ (p ≤ 0.10); these were included in a multivariable Cox model with stepwise selection. In the final model adjusted for other clinical covariates, only four antibody targets, TUBB (HR=2.40; 95% CI 1.37 – 4.21, p=0.002), Collagen III (HR=1.67; 95% CI 1.08 – 2.58, p=0.02), VCL (HR=2.04; 95% CI 1.12 – 3.71, p=0.02) and STAT6 (HR=1.47; 95% CI 1.01 – 2.15, p=0.04) were identified as independent risk factors of ABMR_h_ (**Table S6**).

### Pretransplant Non-HLA Antibodies and Allograft Failure

By univariable Cox regression analysis, the presence of pretransplant non-HLA antibodies, assessed by the broadness and the strength, was not associated with graft failure rates (**Table S7**), also not when confined to the HLA-DSA negative patients (N=774) (**Table 4**). The analysis with the individual pretransplant non-HLA antibodies and stepwise selection approach suggested independent association of antibodies against IYD (HR=2.80; 95% CI 1.08 – 2.14, p=0.009) and NPHS1 (HR=0.27; 95% CI 0.08 – 0.89, p=0.03) with graft failure (**Table S8**). However, when the suggested antibodies were entered together with the clinical covariates, none of the antibodies was independently associated with graft failure in the final Cox model.

**Table 4:** Univariable and multivariable Cox proportional hazards analysis of death-censored graft failure, according to the presence of pretransplant non-HLA antibodies in the absence of HLA-DSA (N=774).

Pretransplant non-HLA antibodies	No. of patients	No. of events	HR	95% CI	p-value
**Univariable analysis**					
***Broadness of non-HLA antibodies***					
**Total positivity for non-HLA antibodies** (per 10)	774	110	0.88	0.69	1.11
**Quartiles for non-HLA abs positivity**	774				
Quartile 1 (0 - 3)	167	31	1	–	–
Quartile 2 (4 - 7)	228	28	0.89	0.45	1.77
Quartile 3 (8 - 14)	174	24	0.84	0.41	1.73
Quartile 4 (15 - 63)	205	27	0.53	0.24	1.16
***Strength of non-HLA antibodies***					
**Total ratios of non-HLA antibodies** (per 10)	774	110	0.98	0.92 – 1.04	0.47
**Quartiles of non-HLA abs strength**	774				
Quartile 1 (0.0 – 9.7)	196	29	1	–	–
Quartile 2 (9.8 – 19.5)	192	28	0.58	0.35 – 0.96	0.03
Quartile 3 (19.6 – 39.5)	191	29	0.70	0.41 – 1.19	0.19
Quartile 4 (39.8 – 223.6)	196	24	0.67	0.40 – 1.12	0.13
**Multivariable analysis**					
***Broadness of non-HLA antibodies***					
**Model 1: Total positivity** (per 10)	774	110	0.84	0.65	1.09
**Model 2: Non-HLA antibodies positivity**	774	110			
Quartile 1 (0 - 3)	167	31	1	–	–
Quartile 2 (4 - 7)	228	28	0.81	0.41	1.63
Quartile 3 (8 - 14)	174	24	0.73	0.35	1.53
Quartile 4 (15 - 63)	205	27	0.43	0.19	0.99
***Strength of non-HLA antibodies***					
**Model 3: Total ratios of non-HLA abs (per 10)**	774	110	0.97	0.91 – 1.03	0.31
**Model 4: Non-HLA antibody strength**	774	110			
Quartile 1 (0.0 – 9.7)	196	29	1	–	–
Quartile 2 (9.8 – 19.5)	192	28	0.57	0.34 – 0.96	0.03
Quartile 3 (19.6 – 39.5)	191	29	0.65	0.65 – 1.11	0.11
Quartile 4 (39.8 – 223.6)	196	24	0.63	0.37 – 1.07	0.08

All multivariable Cox models were adjusted for anti-HLA antibodies, HLA-A,-B,-DR,-DQ antigen mismatches, repeated transplantation, deceased donation, recipient sex, recipient and donor age and induction therapy.

### Posttransplant Non-HLA Antibodies and Histology of Antibody-Mediated Rejection

**Figure 6:**
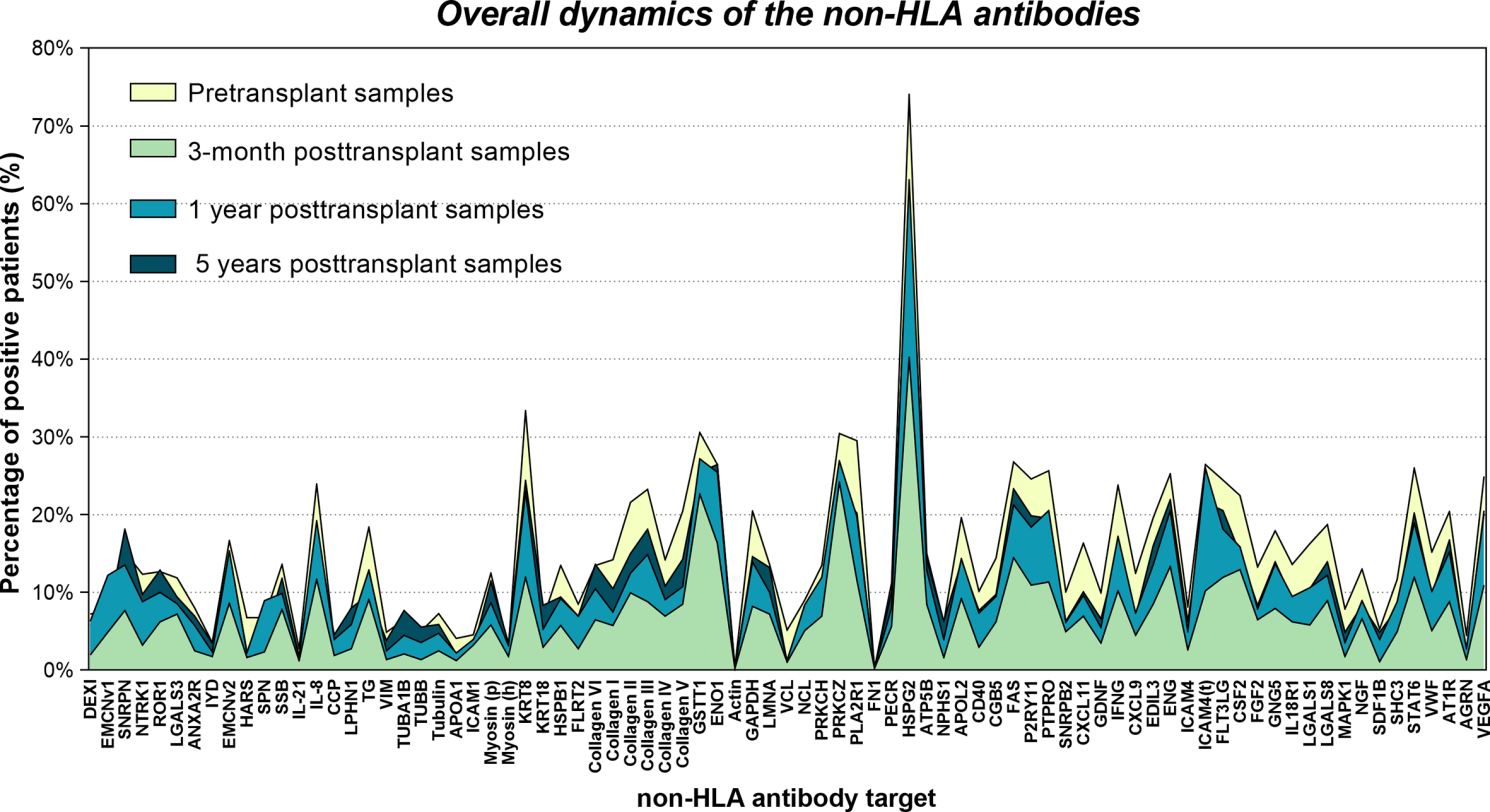
Kinetics of the non-HLA antibodies over time. The graph shows the percentage of positive patients at the day of transplantation (pretransplant), at 3 months, 1 year and 5 years after transplantation.

Finally, we investigated non-HLA antibody reactivity in 1996 post-transplant sera of 876 patients. **Figure 6** depicts the complete kinetics of non-HLA antibodies per individual non-HLA target. There was a global decrease in non-HLA antibody reactivity after kidney transplantation, with the lowest reactivity at 3 months. This reactivity increased again in the samples at 1-year and 5-year samples, but below the pretransplant levels. Paired t tests between the different time points confirmed these kinetics (**Figure S3**).

By comparing the total post-transplant non-HLA antibody reactivity between patients with and without ABMR_h_, the medians of the broadness [4.0 (IQR=2.0-9.0) vs. 4.0 (IQR=2.0-8.0), p=0.15] and the strength [11.6 (IQR=5.2-23.4) vs. 10.5 (IQR=4.6-19.7), p=0.75] were not significantly different between the two groups (**Table 5**). When we compared the total post-transplant non-HLA antibody reactivity at different time points (at 3-month, 1-year, and 5-year) separately, we found no significant difference between the two groups.

**Table 5:** Comparison of the medians of the strength and the broadness of posttransplant non-HLA antibody samples between patients with and without ABMR_h_.

Post-transplant non-HLA antibodies	No ABMR_h_	ABMR_h_	p-value
***Total post-transplant samples***			
**(*N = 1996)***	***N=1766***	***N=230***	
Total broadness of non-HLA antibodies, median (q1-q3)	4.0 (2.0-9.0)	4.0 (2.0-8.0)	0.15
Total strength of non-HLA antibodies, median (q1-q3)	11.6 (5.2 – 23.4)	10.5 (4.6-19.7)	0.31
***Patients at 3-month post-transplant***			
**(*N = 804)***	***N=740***	***N=64***	
Broadness of non-HLA antibodies at 3-month, median (q1-q3)	3.0 (2.0-6.0)	2.0 (2.0-6.5)	0.37
Strength of non-HLA antibodies at 3-month, median (q1-q3)	9.1 (3.4-17.02)	8.0 (3.3-17.4)	0.75
***Patients at 1*-year *post-transplant***			
**(*N = 751)***	***N=691***	***N=60***	
Broadness of non-HLA antibodies at 1-year, median (q1-q3)	5.0 (3.0-12.0)	4.5 (2.0 -9.0)	0.13
Strength of non-HLA antibodies at 1-year, median (q1-q3)	14.1 (7.01 – 28.0)	13.4 (5.4-30.1)	0.53
***Patients at 5*-year *post-transplant***			
**(*N = 287)***	***N=280***	***N=7***	
Broadness of non-HLA antibodies at 5-year, median (q1-q3)	5.0 (3.0-12.0)	3.0 (1.0-5.0)	0.03
Strength of non-HLA antibodies at 5-year, median (q1-q3)	13.4 (6.8-28.0)	4.9 (2.8-13.7)	0.05

ABMR_h_, histology of antibody-mediated rejection.

The medians between the two groups were compared using the Wilcoxon test.

## Discussion

In a large cohort of 934 kidney transplant recipients, this study shows that a high degree of pretransplant non-HLA autoantibody burden is independently associated with increased rates of developing ABMR_h_ in the absence of HLA-DSA. However, the overall posttransplant non-HLA autoreactivity was not associated with increased rates of ABMR_h_, and no associations were observed between the presence of non-HLA autoantibodies and transplant glomerulopathy or graft failure.

ABMR_h_ in the absence of HLA-DSA is a well-recognized phenotype in kidney transplantation, and several studies have suggested the involvement of non-HLA immunity in its development (2, 30–32). However, the clinical role of non-HLA antibody profiling for diagnosing ABMR is still not clear. The present study is the first that implicates the broadness and the strength of the pretransplant autoantibody response against a large panel of non-HLA antigens, which represent a signature of autoreactive response, in the occurrence of ABMR_h_. Furthermore, we found a weak positive correlation between the pretransplant non-HLA autoantibody profile and pretransplant total gamma globulins level; however, the latter did not associate with the development of ABMR_h_. Therefore, this finding supports the hypothesis that the autoimmune responses target specific but larger range of non-HLA antigens.

We showed indeed that high levels of pretransplant non-HLA autoantibodies are independently associated with HLA-DSA negative ABMR_h_. This suggests direct involvement of autoimmunity in developing this histologic phenotype suggestive for ABMR. Additionally, the specific associations with microvascular inflammation (Banff score >2) and glomerulitis (Banff score >1), indicate that the autoantibodies primarily target the glomeruli. Moreover, our data suggested that the non-HLA antibodies could also explain cases of C4d positive ABMR_h_, in the absence of HLA-DSA.

Although our studies primarily suggested broad non-HLA antibody responses in association with histological endpoints, we tried to identify possible individual non-HLA autoantibody associated with ABMR_h_. With the stepwise selection in a multivariable Cox model, we did not find an association between pretransplant anti-AT1R autoantibodies and the occurrence of ABMR_h_. Still, we found four autoantibodies (against TUBB, Collagen III, VCL and STAT6), to associate independently with ABMR_h_ in the absence of HLA-DSA. Although of potential relevance, the exact significance of serum reactivity to a single non-HLA autoantigen and the pathogenic potential still needs to be elucidated. Our present study suggests that the total burden of the pretransplant autoantibodies is more informative than the individual response to a single non-HLA antigen.

It is noteworthy that only ten patients of our cohort did not show elevated reactivity above the threshold MFI to any of the non-HLA autoantibodies in the panel. Hemodialysis was associated independently with the broadness and strength of the non-HLA autoantibody profile. Almost all patients of our cohort (98.7%) showed broad non-HLA autoantibody reactivity, and only 15.1% of them received a previous kidney transplant. This suggests that the antibodies measured in the panel are primarily autoimmune and potentially occurred during the patients’ end-stage renal disease or chronic hemodialysis. Due to the extensive cellular damage and cell death in kidneys with end-stage disease, the patients’ immune system is exposed to many non-HLA targets, potentially resulting in the production of autoantibodies (33). Finally, it needs to be mentioned that there is also a possibility for some of these antibodies to react to multiple, distinct self and exogenous antigenic structures, known as natural antibodies, previously linked to poor kidney graft outcomes (34).

Although the autoantibodies associated with the risk of ABMR_h_, they were not associated with the development of transplant glomerulopathy or graft failure. Even more, only pretransplant and not post-transplant antibodies associated with ABMR_h_. This suggests that these autoantibodies are mainly relevant in early posttransplant phenotypes and do not have a long-lasting impact. As we published earlier, compared to ABMR diagnosed with HLA-DSA positive cases, DSA-negative ABMR_h_ is more transient and associates with better graft survival (3, 35). The non-HLA autoantibodies could potentially play a role in this phenomenon. As we observed a clear lowering of the autoimmune post-transplantation, it appears that the maintenance immunosuppression is sufficient to reduce the level of these autoantibodies, and likely is able to abolish the long-term adverse effect of these autoantibodies on graft outcomes.

The present study has several strengths. We studied a large and well-characterized kidney transplant cohort with longitudinally collected patient serum samples. Using a newly developed multiplex kit, we studied non-HLA antibody response against a large panel of non-HLA antigen targets and changes of the antibody response over time after kidney transplantation. Finally, we integrated the non-HLA antibody data from our cohort with complete histopathologic data. Nevertheless, there are also several limitations to our study that should be considered. First, the study’s generalizability is limited by its retrospective single-center design. Second, for the diagnosis of ABMR_h_, we relied on the diagnostic criteria of the latest Banff classification from 2019; therefore, future updates of the diagnostic criteria for ABMR could impact on the interpretation of our data. Third, we lack data on medication adherence, which could impact kidney graft outcomes; however, it is challenging to assess non-adherence objectively in daily clinical practice. Another limitation is the lack of well characterized clinical samples to assess the performance of each antigen. The performance of each antigen was assessed, by the manufacturer of the beads, using monoclonal antibodies. However the specific performance characteristic of each monoclonal antibody is unknown. It is therefore not possible to evaluate whether the confirmation of the antigens is native, denatured, or a mixture of both. However, the manufacturing process is similar to what is used for the LIFECODES LSA class I, which has been shown to have relatively low levels of denatured antigen (36). In addition, much of the false positive reactivity associated with HLA antibody tests relates to the trimeric state of the HLA antigen – alpha chain, beta chain and peptide, where different dimers or monomers may be found on the bead surface, and reactivity may further be attenuated by the peptide present (37). Many of the antigens identified in this study (STAT6, IYD, NPHS1, TUBB, VCL) are recombinant monomers although incorrect folding cannot be ruled out. While the lack of correlation of any specific antigen with clinical outcome may solely be a reflection of the assay performance of that particular antigen, it is important to note that these products became available only recently, and studies such as outlined herein are critical to understanding the limitations and utility of these assays. Finally, we lack an objective, standardized and clinically relevant cut-off for determining positive results for the autoantibodies, as even healthy individuals potentially have antibodies against some targets. It is important to note that even though the HLA single antigen products have been in use for over a decade, there are still no standardized cutoffs (38). More work is needed on the definition of the thresholds and on the potential relevance of individual antibody levels.

In conclusion, this study indicates that highly and broadly sensitized patients against non-HLA targets are at increased risk of ABMR_h_ after kidney transplantation, in the absence of HLA-DSA. However, no association was found between the broadness and the strength of pretransplant non-HLA autoantibodies and the development of transplant glomerulopathy and graft failure. These findings need to be validated. Whether the individual associations found are clinically relevant and represent causality, warrants further studies.

## Data Availability Statement

All of the individual de-identified participant data that underlie the results reported in this article (text, tables, figures, and appendices) can be made available on a collaborative basis following institutional review board approval. Proposals should be directed at the corresponding author.

## Ethics Statement

The studies involving human participants were reviewed and approved by The Ethics Committee of the University Hospitals Leuven approved this retrospective study (S64006). The patients/participants provided their written informed consent to participate in this study.

## Author Contributions

AS and MN designed the research. BR, JH, and CH performed the non-HLA antibody testing. AS, EL, DK, BS, M-PE, and MN were involved in data collection. AS analyzed the data and performed the statistical analyses. AS and MN wrote the manuscript. All authors revised the manuscript and approved the submitted version.

## Funding

This project is funded by the Research Foundation - Flanders (FWO) and the Flanders Innovation & Entrepreneurship agency (VLAIO), with a TBM (Toegepast Biomedisch onderzoek met een primair Maatschappelijke finaliteit) project (grant n° IWT.150199; “TEMPLATE”). MN is senior clinical investigators of The Research Foundation Flanders (FWO) (1844019N) and is also funded by a C3 internal grant from the KU Leuven University (grant no. C32/17/049). BS is a senior clinical investigator of The Research Foundation Flanders (FWO) (1842919N).

## Conflict of Interest

BR, JH, and CH are employees of Immucor Inc.

The remaining authors declare that the research was conducted in the absence of any commercial or financial relationships that could be construed as a potential conflict of interest.

## Publisher’s Note

All claims expressed in this article are solely those of the authors and do not necessarily represent those of their affiliated organizations, or those of the publisher, the editors and the reviewers. Any product that may be evaluated in this article, or claim that may be made by its manufacturer, is not guaranteed or endorsed by the publisher.
