# Supplementary material for: The Pre-Transplant Non-HLA Antibody Burden Associates With the Development of Histology of Antibody-Mediated Rejection After Kidney Transplantation
Source: Front Immunol. 2022 Feb 16;13:809059. doi: 10.3389/fimmu.2022.809059 (PMC8888449; doi:10.3389/fimmu.2022.809059)

Supplementary Material

**The pre-transplant non-HLA antibody burden associates with the development of histology of antibody-mediated rejection after kidney transplantation.**

Aleksandar Senev, Bryan Ray, Evelyne Lerut, Jayasree Hariharan, Christine Heylen, Dirk Kuypers, Ben Sprangers, Marie-Paule Emonds and Maarten Naesens.

Contents

[***Table S1.*** Antigen list of *Immucor* prototype assay for detection non-HLA antibodies. 2](#_Toc86668350)

[***Table S2.*** Univariable and multivariable Cox analysis for the occurrence of the histologic lesions and phenotypes, according to the broadness of pretransplant non-HLA antibodies (N=874). 4](#_Toc86668351)

[***Table S3.*** Univariable and multivariable Cox proportional hazards analysis for the histologic lesions and phenotypes occurrence, according to the strength of pretransplant non-HLA antibodies (N=874). 5](#_Toc86668352)

[***Table S4.*** Univariable and multivariable Cox analysis for the histologic lesions and phenotypes, according to the strength of the positive pretransplant non-HLA antibodies in the absence of HLA-DSA (N=774). 6](#_Toc86668353)

[**Table S5.** Univariable and multivariable Cox proportional hazards analysis of ABMR_h_, according to the broadness and strength of pretransplant non-HLA antibodies (N=774). 7](#_Toc86668354)

[***Table S6.*** Univariable and multivariable Cox proportional hazards analysis of occurrence of ABMRh in absence of HLA-DSA according to presence of pretransplant non-HLA antibodies (N=774). 8](#_Toc86668355)

[***Table S7.*** Univariable and multivariable Cox proportional hazards analysis of death-censored graft failure; according to the presence of pretransplant non-HLA antibodies (N=874). 10](#_Toc86668356)

[***Table S8.*** Univariable and multivariable Cox proportional hazards analysis of death-censored graft failure, according to the presence of individual pretransplant non-HLA antibodies in the absence of HLA-DSA (N=774). 11](#_Toc86668357)

[***Figure S1.*** Distribution of the A) measured raw MFI values and B) ratios above the cut-off per non-HLA antigen in all pretransplant sera (N=874). 13](#_Toc86668358)

[***Figure S2.*** Pearson correlation matrix between the positive ratios of the antibodies against 81 non-HLA antigens and HLA-DSA. 14](#_Toc86668359)

[***Figure S3.*** Comparison of the raw MFIs values for all antigen targets included in the kit at different time points. 15](#_Toc86668360)

***Table S1.* Antigen list of *Immucor* prototype assay for detection non-HLA antibodies.**

| **Probe** | **Antigen** | **MFI Cutoff** | **Description** |
| --- | --- | --- | --- |
| 1 | Actin | 320 | Actin |
| 2 | AGRN | 1444 | Agrin |
| 3 | ANXA2R | 552 | Annexin A2 receptor |
| 4 | APOA1 | 507 | Apolipoprotein A- I |
| 5 | APOL2 | 635 | Apolipoprotein L, 2 |
| 6 | AT1R | 339 | Angiotensin II Type-1 Receptor |
| 7 | ATP5B | 905 | ATP synthase, H+ transporting, mitochondrial F1 complex, beta polypeptide |
| 8 | CCP | 245 | Cyclic citrullinated peptide |
| 9 | CD40 | 378 | CD40 molecule, TNF receptor superfamily member 5 |
| 10 | CGB5 | 1081 | Chorionic gonadotropin, beta polypeptide 5 |
| 11 | Collagen I | 1204 | Collagen I |
| 12 | Collagen II | 1174 | Collagen II |
| 13 | Collagen III | 1904 | Collagen III |
| 14 | Collagen IV | 651 | Collagen IV |
| 15 | Collagen V | 1015 | Collagen V |
| 16 | Collagen VI | 636 | Collagen VI |
| 17 | CSF2 | 514 | Colony stimulating factor 2 |
| 18 | CXCL11 | 515 | Chemokine (C-X- C motif) ligand 11 |
| 19 | CXCL9 | 565 | C-X-C Motif Chemokine 9 |
| 20 | DEXI | 540 | Dexamethasone- induced transcript |
| 21 | EDIL3 | 462 | EGF-like repeats and discoidin I- like domains 3 |
| 22 | EMCNv1 | 106 | Endomucin |
| 23 | EMCNv2 | 543 | Endomucin |
| 24 | ENG | 252 | Endoglin |
| 25 | ENO1 | 2250 | Alpha-enolase |
| 26 | FAS | 323 | Fas cell surface death receptor |
| 27 | FGF2 | 771 | Fibroblast growth factor receptor 2 |
| 28 | FLRT2 | 949 | Leucine-rich repeat transmembrane protein FLRT2 |
| 29 | FLT3LG | 302 | Fms-related tyrosine kinase 3 ligand |
| 30 | FN1 | 1191 | Fibronectin 1 |
| 31 | GAPDH | 838 | Glyceraldehyde- 3-phosphate dehydrogenase |
| 32 | GDNF | 904 | Glial cell derived neurotrophic factor |
| 33 | GNG5 | 446 | G Protein Subunit Gamma 5 |
| 34 | GSTT1 | 689 | Glutathione S- Transferase theta-1 |
| 35 | HARS | 2704 | Histidyl-tRNA, Jo-1 |
| 36 | HSPB1 | 378 | Heat shock protein beta-1 |
| 37 | HSPG2, Perlecan, LG3 | 662 | Heparan sulfate proteoglycan 2 |
| 38 | ICAM1 | 179 | Intracellular Adhesion Molecule 1 |
| 39 | ICAM4 | 375 | Intercellular adhesion molecule 4 (Landsteiner-Wiener blood group) |
| 40 | ICAM4(t) | 786 | Intercellular adhesion molecule 4 (Landsteiner-Wiener blood group) |
| 41 | IFNG | 443 | Interferon Gamma |
| 42 | IL18R1 | 348 | Interleukin 18 Receptor 1 |
| 43 | IL-21 | 556 | Interleukin 21 |
| 44 | IL-8 | 1262 | Interleukin 8, CXCL8 |
| 45 | IYD | 585 | Iodotyrosine deiodinase |
| 46 | KRT18 | 912 | Cytokeratin 18 |
| 47 | KRT8 | 1366 | Cytokeratin 8 |
| 48 | LGALS1 | 824 | Lectin, galactoside- binding, soluble, 1 |
| 49 | LGALS3 | 508 | lectin, galactoside- binding, soluble, 3 |
| 50 | LGALS8 | 758 | Lectin, galactoside- binding, soluble, 8 |
| 51 | LMNA | 3064 | Prelamin-A/C |
| 52 | LPHN1 | 780 | Latrophilin 1 |
| 53 | MAPK1 | 1341 | Mitogen- Activated Protein Kinase 1 |
| 54 | Native Myosin | 3124 | Myosin, Porcine |
| 55 | Native Myosin | 4491 | Myosin, Human |
| 56 | NCL | 1916 | Nucleolin |
| 57 | NGF | 1266 | Nerve growth factor |
| 58 | NPHS1 | 1025 | Nephrosis 1, congenital, Finnish type (nephrin) |
| 59 | NTRK1 | 408 | Neurotrophic Tyrosine Kinase, Receptor, Type 1 |
| 60 | P2RY11 | 440 | Purinergic receptor P2Y, G- protein coupled, 11 |
| 61 | PECR | 2733 | Peroxisomal trans-2-enoyl- CoA Reductase |
| 62 | PLA2R1 | 637 | Phospholipase A2 receptor 1, 180kDa |
| 63 | PRKCH | 1815 | Protein kinase C, eta |
| 64 | PRKCZ | 2269 | Protein kinase C, zeta |
| 65 | PTPRO | 494 | Receptor-type Tyrosine-protein Phosphatase U |
| 66 | ROR1 | 422 | Receptor Tyrosine Kinase- Like Orphan Receptor 1 |
| 67 | SDF1B | 646 | C-X-C Motif Chemokine Ligand 12 |
| 68 | SHC3 | 1005 | SHC Adaptor Protein 3 |
| 69 | SNRPB2 | 3335 | Small nuclear ribonucleoprotein polypeptide B |
| 70 | SNRPN | 4020 | Small Nuclear Ribonucleoprotein Polypeptide N (smith antigen core sequence) |
| 71 | SPN | 133 | Sialophorin, CD43 |
| 72 | SSB | 3282 | Sjogren syndrome antigen B (autoantigen La) |
| 73 | STAT6 | 1235 | Signal Transducer and Activator of Transcription 6 |
| 74 | TG | 1139 | Thyroglobulin |
| 75 | Transferrin | 572 | Transferrin (negative control) |
| 76 | TUBA1B | 417 | Tubulin, alpha 1b |
| 77 | TUBB | 536 | Tubulin beta |
| 78 | Tubulin | 385 | Tubulin |
| 79 | VCL | 8986 | Vinculin |
| 80 | VEGFA | 567 | Vascular endothelial growth factor A |
| 81 | VIM | 552 | Vimentin |
| 82 | VWF | 456 | Von Willebrand Factor |

EMCNv1 and EMCNv2 are different splice variants; ICAM4(t) is from a transiently expressing cell line.

Abbreviation: MFI, median fluorescence intensity.

***Table S2.* Univariable and multivariable Cox analysis for the occurrence of the** **histologic lesions and phenotypes, according to the broadness of pretransplant non-HLA antibodies (N=874).**

| **Predictor: Broadness of pretransplant non-HLA antibodies** (per 10 antibodies increment) | | | | | | |
| --- | --- | --- | --- | --- | --- | --- |
| **Histologic outcome of the model** | **Univariable analysis** | | | **Multivariable** **analysis** | | |
|  | **HR** | **95%CI** | **p-value** | **HR** | **95%CI** | **p-value** |
| **Histologic phenotypes** (events) |  |  |  |  |  |  |
| ABMR_h_ (204) | 1.12 | 1.01 – 1.23 | **0.03** | 1.14 | 1.03 – 1.27 | 0.01 |
| ABMR 2019 (107) | 0.99 | 0.85 – 1.16 | 0.91 | 1.09 | 0.91 – 1.29 | 0.36 |
| TCMR (273) | 1.01 | 0.92 – 1.11 | 0.88 | 1.01 | 0.92 – 1.11 | 0.86 |
| TCMR + Borderline (403) | 1.04 | 0.96 – 1.12 | 0.37 | 1.03 | 0.95 – 1.11 | 0.45 |
| Microvascular inflammation score ≥ 2 (226) | 1.08 | 0.98 – 1.19 | 0.10 | 1.10 | 0.99 – 1.22 | 0.07 |
| IFTA score 2 (425) | 1.03 | 0.95 – 1.11 | 0.50 | 1.03 | 0.95 – 1.11 | 0.47 |
| **Individual lesions** (events) |  |  |  |  |  |  |
| Glomerulitis (g) score > 0 (284) | 1.04 | 0.95 – 1.14 | 0.37 | 1.04 | 0.95 – 1.15 | 0.38 |
| Glomerulitis (g) score > 1 (129) | 1.05 | 0.92 – 1.20 | 0.46 | 1.05 | 0.91 – 1.21 | 0.55 |
| Peritubular capillaritis (ptc) score> 0 (293) | 1.00 | 0.91 – 1.09 | 0.92 | 1.00 | 0.91 – 1.11 | 0.93 |
| Peritubular capillaritis (ptc) score> 1 (146) | 1.01 | 0.89 – 1.15 | 0.89 | 1.04 | 0.91 – 1.18 | 0.59 |
| Endarteritis (v) score> 0 (190) | 1.04 | 0.93 – 1.16 | 0.48 | 1.05 | 0.94 – 1.18 | 0.41 |
| Endarteritis (v) score> 1 (27) | 1.07 | 0.81 – 1.42 | 0.65 | 1.11 | 0.83 – 1.47 | 0.48 |
| c4d score > 0 (334) | 1.00 | 0.92 – 1.09 | 0.97 | 0.99 | 0.91 – 1.09 | 0.89 |
| c4d score > 1 (101) | 1.03 | 0.88 – 1.20 | 0.73 | 1.06 | 0.90 – 1.24 | 0.50 |
| Interstitial inflammation (i) score> 0 (383) | 1.02 | 0.94 – 1.10 | 0.63 | 1.01 | 0.93 – 1.10 | 0.81 |
| Interstitial inflammation (i) score> 1 (229) | 1.03 | 0.93 – 1.14 | 0.54 | 1.03 | 0.93 – 1.14 | 0.57 |
| Tubulitis (t) score> 0 (616) | 1.01 | 0.95 – 1.07 | 0.82 | 0.99 | 0.93 – 1.05 | 0.71 |
| Tubulitis (t) score> 1 (298) | 1.05 | 0.96 – 1.15 | 0.26 | 1.02 | 0.93 – 1.11 | 0.71 |
| Chronic allograft glomerulopathy (cg) score>0 (93) | 0.82 | 0.66 – 1.01 | 0.06 | 0.79 | 0.64 – 0.99 | 0.04 |
| Chronic allograft glomerulopathy (cg) score>1 (45) | 0.78 | 0.57 – 1.06 | 0.12 | 0.78 | 0.56 – 1.08 | 0.14 |
| Arteriolar hyalinosis (ah) score> 1 (298) | 1.02 | 0.93 – 1.18 | 0.73 | 1.00 | 0.91 – 1.10 | 0.99 |
| Interstitial fibrosis (ci) score> 1 (418) | 1.02 | 0.95 – 1.10 | 0.61 | 1.02 | 0.95 – 1.11 | 0.56 |
| Tubular atrophy (ct) score> 1 (341) | 1.01 | 0.93 – 1.10 | 0.83 | 1.01 | 0.92 – 1.11 | 0.83 |
| Vascular intimal thickening (cv) score> 1 (411) | 1.03 | 0.95 – 1.11 | 0.48 | 1.00 | 0.92 – 1.08 | 0.93 |
| Mesangial matrix expansion (mm) score> 0 (221) | 1.03 | 0.93 – 1.15 | 0.58 | 1.00 | 0.89 – 1.11 | 0.95 |

All multivariable Cox models were adjusted for HLA-DSA, HLA-A, -B, -DR, -DQ antigen mismatches, repeated transplantation, deceased donation, recipient sex, recipient and donor age and induction therapy.

***Table S3.* Univariable and multivariable Cox proportional hazards analysis for the histologic lesions and phenotypes occurrence, according to the strength of pretransplant non-HLA antibodies (N=874).**

| **Predictor: Strength of pretransplant non-HLA antibodies** (per 10 ratios increment) | | | | | | |
| --- | --- | --- | --- | --- | --- | --- |
| **Histologic outcome of the model** | **Univariable analysis** | | | **Multivariable** **analysis** | | |
|  | **HR** | **95%CI** | **p-value** | **HR** | **95%CI** | **p-value** |
| **Histologic phenotypes** (events) |  |  |  |  |  |  |
| ABMR_h_ (204) | 1.05 | 1.01 – 1.09 | 0.01 | 1.05 | 1.01 – 1.09 | 0.009 |
| ABMR 2019 (107) | 1.02 | 0.96 – 1.07 | 0.57 | 1.05 | 0.99 – 1.12 | 0.14 |
| TCMR (273) | 1.01 | 0.98 – 1.05 | 0.54 | 1.01 | 0.97 – 1.05 | 0.60 |
| TCMR + Borderline (403) | 1.02 | 0.99 – 1.05 | 0.16 | 1.02 | 0.99 – 1.05 | 0.24 |
| Microvascular inflammation score ≥2 (226) | 1.04 | 1.00 – 1.07 | 0.05 | 1.04 | 1.00 – 1.08 | 0.05 |
| IFTA score 2 (425) | 1.03 | 1.00 – 1.06 | 0.07 | 1.02 | 0.99 – 1.05 | 0.12 |
| **Individual lesions** (events) |  |  |  |  |  |  |
| Glomerulitis (g) score > 0 (284) | 1.02 | 0.99 – 1.05 | 0.27 | 1.02 | 0.98 – 1.06 | 0.30 |
| Glomerulitis (g) score > 1 (129) | 1.02 | 0.98 – 1.07 | 0.50 | 1.01 | 0.96 – 1.07 | 0.70 |
| Peritubular capillaritis (ptc) score> 0 (293) | 1.02 | 0.98 – 1.05 | 0.40 | 1.02 | 0.98 – 1.05 | 0.37 |
| Peritubular capillaritis (ptc) score> 1 (146) | 1.01 | 0.97 – 1.06 | 0.60 | 1.02 | 0.97 – 1.07 | 0.49 |
| Endarteritis (v) score> 0 (190) | 1.03 | 0.99 – 1.07 | 0.13 | 1.03 | 0.99 – 1.08 | 0.12 |
| Endarteritis (v) score> 1 (27) | 1.03 | 0.92 – 1.14 | 0.64 | 1.04 | 0.94 – 1.16 | 0.45 |
| c4d score > 0 (334) | 1.01 | 0.98 – 1.05 | 0.49 | 1.01 | 0.98 – 1.04 | 0.64 |
| c4d score > 1 (101) | 1.03 | 0.97 – 1.08 | 0.37 | 1.04 | 0.98 – 1.10 | 0.26 |
| Interstitial inflammation (i) score> 0 (383) | 1.01 | 0.98 – 1.05 | 0.40 | 1.01 | 0.98 – 1.04 | 0.51 |
| Interstitial inflammation (i) score> 1 (229) | 1.01 | 0.98 – 1.05 | 0.47 | 1.01 | 0.97 – 1.05 | 0.55 |
| Tubulitis (t) score> 0 (616) | 1.00 | 0.98 – 1.03 | 0.76 | 0.99 | 0.97 – 1.02 | 0.63 |
| Tubulitis (t) score> 1 (298) | 1.01 | 0.98 – 1.05 | 0.41 | 1.00 | 0.97 – 1.04 | 0.94 |
| Chronic allograft glomerulopathy (cg) score>0 (93) | 0.92 | 0.85 – 1.01 | 0.07 | 0.91 | 0.83 – 0.99 | 0.04 |
| Chronic allograft glomerulopathy (cg) score>1 (45) | 0.89 | 0.78 – 1.02 | 0.10 | 0.86 | 0.77 – 1.02 | 0.09 |
| Arteriolar hyalinosis (ah) score> 1 (298) | 1.00 | 0.96 – 1.04 | 0.86 | 0.99 | 0.95 – 1.02 | 0.44 |
| Interstitial fibrosis (ci) score> 1 (418) | 1.02 | 0.99 – 1.05 | 0.11 | 1.02 | 0.99 – 1.05 | 0.16 |
| Tubular atrophy (ct) score> 1 (341) | 1.02 | 0.99 – 1.05 | 0.28 | 1.02 | 0.98 – 1.05 | 0.38 |
| Vascular intimal thickening (cv) score> 1 (411) | 1.02 | 0.99 – 1.05 | 0.27 | 1.00 | 0.97 – 1.03 | 0.95 |
| Mesangial matrix expansion (mm) score> 0 (221) | 0.99 | 0.95 – 1.04 | 0.84 | 0.98 | 0.94 – 1.03 | 0.37 |

All multivariable Cox models were adjusted for HLA-DSA, HLA-A, -B, -DR, -DQ antigen MM, repeated transplantation, deceased donation, recipient sex, recipient and donor age and induction therapy.

***Table S4.* Univariable and multivariable Cox analysis for the histologic lesions and phenotypes, according to the strength of the positive pretransplant non-HLA antibodies in the absence of HLA-DSA (N=774).**

| **Predictor: Strength of pretransplant non-HLA antibodies** (per 10 ratios increment) |  |  |  |  |  |  |
| --- | --- | --- | --- | --- | --- | --- |
| **Histologic outcome of the model** | **Univariable** **analysis** | | | **Multivariable** **analysis** | | |
|  | **HR** | **95%CI** | **p-value** | **HR** | **95%CI** | **p-value** |
| **Histologic phenotypes** (events) |  |  |  |  |  |  |
| ABMR_h_ (125) | 1.07 | 1.03 – 1.12 | 0.001 | 1.06 | 1.02 – 1.11 | 0.005 |
| ABMR 2019 (29) | 1.09 | 1.00 – 1.18 | 0.04 | 1.09 | 1.02 – 1.18 | 0.04 |
| TCMR (218) | 1.01 | 0.97 – 1.05 | 0.57 | 1.01 | 0.97 – 1.05 | 0.69 |
| TCMR and borderline (330) | 1.02 | 0.99 – 1.05 | 0.20 | 1.02 | 0.98 – 1.05 | 0.33 |
| Microvascular inflammation score ≥2 (148) | 1.05 | 1.01 – 1.09 | 0.02 | 1.04 | 1.00 – 1.09 | 0.06 |
| IFTA score 2 (425) | 1.02 | 0.99 – 1.05 | 0.16 | 1.02 | 0.99 – 1.05 | 0.23 |
| **Individual lesions** (events) |  |  |  |  |  |  |
| Glomerulitis (g) score> 0 (203) | 1.03 | 0.99 – 1.07 | 0.17 | 1.02 | 0.98 – 1.06 | 0.33 |
| Glomerulitis (g) score> 1 (70) | 1.06 | 1.00 – 1.12 | 0.08 | 1.04 | 0.98 – 1.11 | 0.20 |
| Peritubular capillaritis (ptc) score> 0 (214) | 1.02 | 0.99 – 1.06 | 0.24 | 1.04 | 0.98 – 1.06 | 0.33 |
| Peritubular capillaritis (ptc) score> 1 (95) | 1.05 | 0.99 – 1.10 | 0.10 | 1.04 | 0.99 – 1.10 | 0.16 |
| Endarteritis (v) score> 0 (139) | 1.03 | 0.99 – 1.08 | 0.18 | 1.02 | 0.98 – 1.07 | 0.34 |
| Endarteritis (v) score> 1 (20) | 1.05 | 0.95 – 1.17 | 0.33 | 1.06 | 0.95 – 1.18 | 0.30 |
| c4d score> 0 (262) | 1.02 | 0.99 – 1.06 | 0.21 | 1.02 | 0.98 – 1.05 | 0.36 |
| c4d score> 1 (50) | 1.07 | 1.00 – 1.14 | 0.06 | 1.07 | 1.00 – 1.14 | 0.07 |
| Interstitial inflammation (i) score> 0 (317) | 1.01 | 0.98 – 1.05 | 0.46 | 1.01 | 0.97 – 1.04 | 0.68 |
| Interstitial inflammation (i) score> 1 (184) | 1.02 | 0.97 – 1.06 | 0.48 | 1.01 | 0.97 – 1.05 | 0.63 |
| Tubulitis (t) score> 0 (531) | 1.00 | 0.98 – 1.03 | 0.89 | 0.99 | 0.96 – 1.02 | 0.49 |
| Tubulitis (t) score> 1 (262) | 1.02 | 0.98 – 1.05 | 0.38 | 1.00 | 0.97 – 1.04 | 0.91 |
| Chronic allograft glomerulopathy (cg) score>0 (70) | 0.94 | 0.86 – 1.03 | 0.20 | 0.93 | 0.84 – 1.02 | 0.14 |
| Chronic allograft glomerulopathy (cg) score>1 (25) | 0.92 | 0.78 – 1.09 | 0.33 | 0.92 | 0.78 – 1.08 | 0.30 |
| Arteriolar hyalinosis (ah) score> 1 (253) | 1.00 | 0.96 – 1.04 | 0.84 | 0.99 | 0.95 – 1.03 | 0.49 |
| Interstitial fibrosis (ci) score> 1 (361) | 1.02 | 0.99 – 1.05 | 0.22 | 1.02 | 0.99 – 1.05 | 0.30 |
| Tubular atrophy (ct) score> 1 (296) | 1.01 | 0.98 – 1.05 | 0.57 | 1.01 | 0.97 – 1.05 | 0.60 |
| Vascular intimal thickening (cv) score> 1 (350) | 1.01 | 0.98 – 1.04 | 0.51 | 1.00 | 0.96 – 1.03 | 0.77 |
| Mesangial matrix expansion (mm) score> 0 (183) | 1.01 | 0.97 – 1.06 | 0.68 | 1.00 | 0.95 – 1.04 | 0.83 |

All multivariable Cox models were adjusted for anti-HLA antibodies, HLA-A, -B, -DR, -DQ antigen mismatch, repeated transplantation, deceased donation, recipient sex, recipient and donor age and induction therapy.

Abbreviation: C4d, complement 4d deposition.

***Table S5.* Univariable and multivariable Cox proportional hazards analysis of ABMR_h_, according to the broadness and strength of pretransplant non-HLA antibodies (N=774).**

| **Pretransplant non-HLA antibodies** | **No. of patients** | **No. of events** | **HR** | **95% CI** | **p-value** |
| --- | --- | --- | --- | --- | --- |
| **Univariable analysis** |  |  |  |  |  |
| ***Broadness of non-HLA antibodies*** |  |  |  |  |  |
| **Quartiles for non-HLA abs positivity** | 774 | 125 |  |  |  |
| Quartile 1 (0 - 3) | 167 | 18 | 1 | - | - |
| Quartile 2 (4 - 7) | 228 | 33 | 1.29 | 0.73 – 2.29 | 0.38 |
| Quartile 3 (8 - 14) | 174 | 25 | 1.26 | 0.68 – 2.33 | 0.46 |
| Quartile 4 (15 - 63) | 205 | 50 | 2.37 | 1.38 – 4.06 | 0.002 |
| ***Strength of non-HLA antibodies*** |  |  |  |  |  |
| **Quartiles of non-HLA abs strength** | 774 | 125 |  |  |  |
| Quartile 1 (0.0 – 9.7) | 196 | 21 | 1 | - | - |
| Quartile 2 (9.8 – 19.5) | 192 | 28 | 1.35 | 0.77 – 2.38 | 0.30 |
| Quartile 3 (19.6 – 39.5) | 191 | 27 | 1.40 | 0.79 – 2.47 | 0.25 |
| Quartile 4 (39.8 – 223.6) | 196 | 49 | 2.67 | 1.54 – 4.28 | 0.0003 |
| ***Total gamma globulins g/L (per 1)*** | 774 | 125 | 1.03 | 0.97 – 1.08 | 0.38 |
| **Multivariable** **analysis** |  |  |  |  |  |
| ***Model 1: Broadness of non-HLA antibodies*** |  |  |  |  |  |
| **Quartiles for non-HLA abs positivity** | 774 | 125 |  |  |  |
| Quartile 1 (0 - 3) | 167 | 18 | 1 | - | - |
| Quartile 2 (4 - 7) | 228 | 33 | 1.31 | 0.74 – 2.34 | 0.36 |
| Quartile 3 (8 - 14) | 174 | 25 | 1.27 | 0.68 – 2.34 | 0.45 |
| Quartile 4 (15 - 63) | 205 | 50 | 2.36 | 1.36 – 4.09 | 0.002 |
| ***Model 2: Strength of non-HLA antibodies*** |  |  |  |  |  |
| **Quartiles of non-HLA abs strength** | 774 | 125 |  |  |  |
| Quartile 1 (0.0 – 9.7) | 196 | 21 | 1 | - | - |
| Quartile 2 (9.8 – 19.5) | 192 | 28 | 1.30 | 0.74 – 2.30 | 0.36 |
| Quartile 3 (19.6 – 39.5) | 191 | 27 | 1.37 | 0.77 – 2.42 | 0.29 |
| Quartile 4 (39.8 – 223.6) | 196 | 49 | 2.54 | 1.51 – 4.27 | 0.0005 |

All multivariable Cox models were adjusted for anti-HLA antibodies, HLA-A, -B, -DR, -DQ antigen mismatches, repeated transplantation, deceased donation, recipient sex, recipient and donor age and induction therapy.

***Table S6.* Univariable and multivariable Cox proportional hazards analysis of occurrence of ABMRh in absence of HLA-DSA according to presence of pretransplant non-HLA antibodies (N=774).**

| **Predictor** | **No. of positive patients** | **HR** | **95% CI** | | **p-value** |
| --- | --- | --- | --- | --- | --- |
| **Univariable Cox analysis** |  |  |  |  |  |
| Actin | 4 | - | - | - | - |
| AGRN | 34 | 1.3 | 0.87 | 1.94 | 0.20 |
| ANXA2R | 65 | 1.64 | 1.31 | 2.06 | **<0.0001** |
| APOA1 | 35 | 1.12 | 0.88 | 1.43 | 0.37 |
| APOL2 | 160 | 1.08 | 0.96 | 1.2 | 0.20 |
| AT1R | 165 | 1.09 | 0.94 | 1.27 | 0.26 |
| ATP5B | 122 | 0.98 | 0.88 | 1.1 | 0.75 |
| CCP | 31 | 1.02 | 0.96 | 1.07 | 0.60 |
| CD40 | 84 | 1.32 | 0.97 | 1.79 | **0.07** |
| CGB5 | 114 | 1.12 | 0.99 | 1.27 | **0.06** |
| Collagen I | 111 | 1.09 | 0.99 | 1.2 | **0.08** |
| Collagen II | 172 | 1.07 | 0.99 | 1.14 | **0.08** |
| Collagen III | 182 | 1.15 | 1.03 | 1.29 | **0.01** |
| Collagen IV | 110 | 1.03 | 0.95 | 1.12 | 0.44 |
| Collagen V | 165 | 1.06 | 0.97 | 1.16 | 0.20 |
| Collagen VI | 103 | 1.03 | 0.94 | 1.13 | 0.55 |
| CSF2 | 178 | 1.04 | 1.01 | 1.08 | **0.01** |
| CXCL11 | 132 | 1.02 | 0.87 | 1.19 | 0.84 |
| CXCL9 | 101 | 1.05 | 0.93 | 1.19 | 0.41 |
| DEXI | 58 | 1.58 | 1.23 | 2.02 | **0.0004** |
| EDIL3 | 159 | 1.08 | 0.9 | 1.3 | 0.39 |
| EMCNv1 | 65 | 1 | 0.96 | 1.05 | 0.91 |
| EMCNv2 | 132 | 1.06 | 0.98 | 1.16 | 0.16 |
| ENG | 202 | 1.01 | 0.92 | 1.1 | 0.87 |
| ENO1 | 203 | 1.05 | 0.95 | 1.16 | 0.36 |
| FAS | 219 | 0.99 | 0.92 | 1.07 | 0.83 |
| FGF2 | 104 | 1.03 | 0.92 | 1.15 | 0.62 |
| FLRT2 | 72 | 1.06 | 0.9 | 1.24 | 0.51 |
| FLT3LG | 201 | 1.05 | 0.98 | 1.12 | 0.16 |
| FN1 | 2 | - | - | - | - |
| GAPDH | 158 | 1.07 | 1 | 1.16 | **0.07** |
| GDNF | 76 | 1.27 | 1.13 | 1.43 | **<0.0001** |
| GNG5 | 142 | 1.08 | 0.93 | 1.24 | 0.31 |
| GSTT1 | 233 | 1.04 | 0.99 | 1.09 | **0.10** |
| HARS (Jo-1) | 52 | 1.12 | 0.83 | 1.5 | 0.47 |
| HSPB1 | 108 | 1.04 | 0.98 | 1.1 | 0.18 |
| ICAM1 | 35 | 1.01 | 0.9 | 1.14 | 0.84 |
| ICAM4 | 66 | 0.98 | 0.68 | 1.4 | 0.91 |
| ICAM4(t) | 211 | 1.27 | 1.1 | 1.46 | **0.001** |
| IFNG | 195 | 1.02 | 0.97 | 1.07 | 0.47 |
| IL18R1 | 114 | 1.14 | 0.94 | 1.39 | 0.20 |
| IL-21 | 20 | - | - | - | - |
| IL-8 | 183 | 1.01 | 0.96 | 1.06 | 0.74 |
| IYD | 28 | 1.38 | 0.99 | 1.94 | **0.06** |
| KRT18 | 53 | 0.95 | 0.75 | 1.21 | 0.68 |
| KRT8 | 260 | 1.02 | 0.94 | 1.09 | 0.69 |
| LGALS1 | 128 | 1.04 | 0.93 | 1.17 | 0.46 |
| LGALS3 | 95 | 0.96 | 0.86 | 1.06 | 0.42 |
| LGALS8 | 151 | 1.03 | 0.91 | 1.15 | 0.69 |
| LMNA | 106 | 1.17 | 0.96 | 1.14 | 0.12 |
| LPHN1 | 61 | 0.92 | 0.67 | 1.27 | 0.60 |
| MAPK1 | 61 | 1.16 | 0.85 | 1.59 | 0.36 |
| Myosin (human) | 25 | 1.36 | 0.96 | 1.92 | **0.08** |
| Myosin (porcine) | 99 | 1.3 | 1.05 | 1.61 | **0.02** |
| NCL | 74 | 1.08 | 0.89 | 1.32 | 0.42 |
| NGF | 106 | 1.12 | 0.96 | 1.31 | 0.14 |
| NPHS1 | 49 | 1.55 | 1.15 | 2.09 | **0.004** |
| NTRK1 | 101 | 1.58 | 1.29 | 1.95 | **<0.0001** |
| P2RY11 | 197 | 1.12 | 0.96 | 1.3 | 0.14 |
| PECR | 87 | 1.14 | 0.94 | 1.38 | 0.17 |
| Perlecan, HSPG2 | 575 | 1.01 | 0.99 | 1.04 | 0.39 |
| PLA2R1 | 238 | 1.01 | 0.94 | 1.08 | 0.89 |
| PRKCH | 109 | 1.03 | 0.89 | 1.2 | 0.70 |
| PRKCZ | 243 | 1.02 | 0.94 | 1.1 | 0.61 |
| PTPRO | 205 | 1.12 | 0.97 | 1.3 | 0.13 |
| ROR1 | 102 | 1.03 | 0.98 | 1.08 | 0.28 |
| SDF1B | 41 | 1.16 | 0.81 | 1.68 | 0.42 |
| SHC3 | 91 | 1.14 | 1.01 | 1.28 | **0.04** |
| SNRPB2 | 77 | 0.98 | 0.71 | 1.37 | 0.92 |
| SNRPN | 122 | 1 | 0.81 | 1.23 | 0.98 |
| SPN | 52 | 1.08 | 0.82 | 1.42 | 0.59 |
| SSB | 106 | 1.09 | 0.91 | 1.31 | 0.33 |
| STAT6 | 211 | 1.13 | 1.05 | 1.22 | **0.002** |
| TG | 143 | 1.03 | 0.96 | 1.1 | 0.43 |
| TUBA1B | 56 | 1.23 | 1.08 | 1.4 | **0.002** |
| TUBB | 41 | 1.17 | 1.01 | 1.37 | **0.04** |
| Tubulin (native) | 59 | 1.11 | 0.97 | 1.26 | 0.13 |
| VCL | 40 | 2.01 | 1.33 | 3.04 | **0.0009** |
| VEGFA | 197 | 1.15 | 0.98 | 1.33 | **0.08** |
| VIM | 43 | 1.09 | 0.91 | 1.31 | 0.37 |
| VWF | 123 | 1.26 | 1.04 | 1.54 | **0.02** |
| **Final multivariable** **analysis**  (Stepwise selection) |  |  |  |  |  |
| TUBB | 41 | 2.40 | 1.37 | 4.21 | 0.002 |
| Collagen III | 182 | 1.67 | 1.08 | 2.58 | 0.02 |
| VCL | 40 | 2.04 | 1.12 | 3.71 | 0.02 |
| STAT6 | 211 | 1.47 | 1.01 | 2.15 | 0.04 |

All variables with P value of ≤0.10, indicated in bold font, from the univariable analysis were included in one multivariable Cox model. The final multivariable Cox model was adjusted for presence of anti-HLA antibodies, HLA-A, -B, -DR, -DQ antigen mismatches, repeat transplantation, deceased donation, recipient sex, recipient and donor age and induction therapy. Abbreviation: see *Table S1*.

***Table S7.* Univariable and multivariable Cox proportional hazards analysis of death-censored graft failure; according to the presence of pretransplant non-HLA antibodies (N=874).**

| **Pretransplant non-HLA antibodies** | **No. of patients** | **No. of events** | **HR** | **95% CI** | **p-value** |
| --- | --- | --- | --- | --- | --- |
| **Univariable analysis** |  |  |  |  |  |
| ***Broadness of non-HLA antibodies*** |  |  |  |  |  |
| **Total positivity for non-HLA abs (per 10)** | 874 | 146 | 0.90 | 1.16 | 0.16 |
| **Quartiles for non-HLA abs positivity** | 874 | 146 |  |  |  |
| Quartile 1 (0 - 3) | 187 | 35 | 1 | - | - |
| Quartile 2 (4 - 7) | 260 | 41 | 0.77 | 0.49 – 1.21 | 0.26 |
| Quartile 3 (8 - 14) | 205 | 37 | 0.94 | 0.59 – 1.49 | 0.79 |
| Quartile 4 (15 - 63) | 222 | 33 | 0.76 | 0.47 – 1.22 | 0.25 |
| ***Strength of non-HLA antibodies*** |  |  |  |  |  |
| **Total ratios of non-HLA abs (per 10)** | 874 | 146 | 0.97 | 0.92 – 1.02 | 0.25 |
| **Quartiles of non-HLA abs strength** | 874 | 146 |  |  |  |
| Quartile 1 (0.0 – 9.7) | 218 | 35 | 1 | - | - |
| Quartile 2 (9.8 – 19.5) | 219 | 39 | 1.15 | 0.73 – 1.82 | 0.55 |
| Quartile 3 (19.6 – 39.6) | 219 | 41 | 1.24 | 0.79 – 1.94 | 0.35 |
| Quartile 4 (39.7 – 223.6) | 218 | 31 | 0.92 | 0.57 – 1.49 | 0.72 |

All multivariable Cox models were adjusted for anti-HLA antibodies, HLA-A, -B, -DR, -DQ antigen MM, repeated transplantation, deceased donation, recipient sex, recipient and donor age and induction therapy.

***Table S8.* Univariable and multivariable Cox proportional hazards analysis of death-censored graft failure, according to the presence of individual pretransplant non-HLA antibodies in the absence of HLA-DSA (N=774).**

| **Antibody** | **No. of positive patients** | **HR** | **95% CI** | | **p-value** |
| --- | --- | --- | --- | --- | --- |
| **Univariable analysis** |  |  |  |  |  |
| Actin | 4 | - | - | - | - |
| AGRN | 34 | 1.64 | 0.76 | 3.52 | 0.21 |
| ANXA2R | 65 | 0.71 | 0.33 | 1.52 | 0.38 |
| APOA1 | 35 | 0.34 | 0.08 | 1.38 | 0.13 |
| APOL2 | 160 | 0.86 | 0.53 | 1.39 | 0.53 |
| AT1R | 165 | 0.77 | 0.47 | 1.25 | 0.29 |
| ATP5B | 122 | 1.22 | 0.75 | 1.99 | 0.41 |
| CCP | 31 | 1.18 | 0.52 | 2.69 | 0.70 |
| CD40 | 84 | 0.95 | 0.51 | 1.76 | 0.86 |
| CGB5 | 114 | 0.66 | 0.35 | 1.23 | 0.19 |
| Collagen I | 111 | 0.98 | 0.58 | 1.66 | 0.94 |
| Collagen II | 172 | 0.74 | 0.45 | 1.22 | 0.24 |
| Collagen III | 182 | 0.73 | 0.45 | 1.17 | 0.18 |
| Collagen IV | 110 | 1.02 | 0.6 | 1.73 | 0.95 |
| Collagen V | 165 | 0.87 | 0.54 | 1.41 | 0.58 |
| Collagen VI | 103 | 0.79 | 0.43 | 1.44 | 0.44 |
| CSF2 | 178 | 0.61 | 0.37 | 1.03 | **0.06** |
| CXCL11 | 132 | 0.89 | 0.53 | 1.49 | 0.65 |
| CXCL9 | 101 | 0.82 | 0.46 | 1.47 | 0.52 |
| DEXI | 58 | 0.98 | 0.48 | 2.01 | 0.95 |
| EDIL3 | 159 | 0.78 | 0.48 | 1.31 | 0.35 |
| EMCNv1 | 65 | 0.98 | 0.49 | 1.94 | 0.96 |
| EMCNv2 | 132 | 0.72 | 0.41 | 1.27 | 0.26 |
| ENG | 202 | 0.86 | 0.55 | 1.34 | 0.50 |
| ENO1 | 203 | 0.94 | 0.61 | 1.46 | 0.80 |
| FAS | 219 | 0.89 | 0.58 | 1.36 | 0.58 |
| FGF2 | 104 | 0.87 | 0.49 | 1.55 | 0.63 |
| FLRT2 | 72 | 0.73 | 0.36 | 1.5 | 0.39 |
| FLT3LG | 201 | 0.81 | 0.52 | 1.28 | 0.37 |
| FN1 | 2 | - | - | - | - |
| GAPDH | 158 | 1.08 | 0.68 | 1.71 | 0.74 |
| GDNF | 76 | 0.65 | 0.32 | 1.34 | 0.23 |
| GNG5 | 142 | 0.91 | 0.55 | 1.5 | 0.70 |
| GSTT1 | 233 | 0.74 | 0.48 | 1.15 | 0.18 |
| HARS (Jo-1) | 52 | 1.47 | 0.76 | 2.81 | 0.25 |
| HSPB1 | 108 | 1.02 | 0.59 | 1.76 | 0.93 |
| ICAM1 | 35 | 0.78 | 0.29 | 2.13 | 0.63 |
| ICAM4 | 66 | 0.75 | 0.35 | 1.62 | 0.47 |
| ICAM4(t) | 211 | 0.9 | 0.59 | 1.39 | 0.64 |
| IFNG | 195 | 0.65 | 0.4 | 1.06 | **0.08** |
| IL18R1 | 114 | 0.66 | 0.35 | 1.23 | 0.19 |
| IL-21 | 20 | 0.33 | 0.05 | 2.39 | 0.28 |
| IL-8 | 183 | 0.83 | 0.52 | 1.33 | 0.44 |
| IYD | 28 | 1.06 | 1 | 4.23 | **0.05** |
| KRT18 | 53 | 1.27 | 0.66 | 2.43 | 0.47 |
| KRT8 | 260 | 1.26 | 0.86 | 1.84 | 0.24 |
| LGALS1 | 128 | 0.81 | 0.47 | 1.39 | 0.44 |
| LGALS3 | 95 | 0.66 | 0.64 | 1.26 | 0.21 |
| LGALS8 | 151 | 0.81 | 0.48 | 1.33 | 0.39 |
| LMNA | 106 | 0.98 | 0.56 | 1.71 | 0.93 |
| LPHN1 | 61 | 0.9 | 0.42 | 1.95 | 0.80 |
| MAPK1 | 61 | 0.93 | 0.45 | 1.91 | 0.84 |
| Myosin (human) | 25 | 0.96 | 0.31 | 3.08 | 0.97 |
| Myosin (porcine) | 99 | 0.88 | 0.48 | 1.61 | 0.68 |
| NCL | 74 | 1.2 | 0.67 | 1.14 | 0.54 |
| NGF | 106 | 0.66 | 0.36 | 1.24 | 0.20 |
| NPHS1 | 49 | 0.37 | 0.12 | 1.16 | **0.09** |
| NTRK1 | 101 | 0.72 | 0.39 | 1.35 | 0.31 |
| P2RY11 | 197 | 0.91 | 0.59 | 1.42 | 0.68 |
| PECR | 87 | 0.77 | 0.4 | 1.48 | 0.44 |
| Perlecan | 575 | 0.89 | 0.59 | 1.36 | 0.59 |
| PLA2R1 | 238 | 0.91 | 0.6 | 1.38 | 0.66 |
| PRKCH | 109 | 1.33 | 0.8 | 2.2 | 0.27 |
| PRKCZ | 243 | 0.79 | 0.51 | 1.21 | 0.27 |
| PTPRO | 205 | 0.92 | 0.59 | 1.41 | 0.68 |
| ROR1 | 102 | 1.09 | 0.64 | 1.84 | 0.76 |
| SDF1B | 41 | 0.96 | 0.42 | 1.18 | 0.92 |
| SHC3 | 91 | 0.72 | 0.38 | 1.38 | 0.33 |
| SNRPB2 | 77 | 0.91 | 0.47 | 1.73 | 0.75 |
| SNRPN | 122 | 1.26 | 0.78 | 2.04 | 0.35 |
| SPN | 52 | 0.43 | 0.14 | 1.36 | 0.15 |
| SSB | 106 | 0.76 | 0.41 | 1.38 | 0.36 |
| STAT6 | 211 | 0.84 | 0.55 | 1.31 | 0.46 |
| TG | 143 | 1.02 | 0.63 | 1.68 | 0.93 |
| TUBA1B | 56 | 0.7 | 0.29 | 1.53 | 0.34 |
| TUBB | 41 | 1.01 | 0.44 | 2.29 | 0.99 |
| Tubulin (native) | 59 | 0.98 | 0.48 | 2.01 | 0.95 |
| VCL | 40 | 0.91 | 0.4 | 2.06 | 0.81 |
| VEGFA | 197 | 0.85 | 0.54 | 1.32 | 0.47 |
| VIM | 43 | 0.55 | 0.2 | 1.51 | 0.25 |
| VWF | 123 | 0.91 | 0.53 | 1.54 | 0.72 |
| **Multivariable** **analysis** |  |  |  |  |  |
| IYD | 28 | 2.80 | 1.08 | 2.14 | 0.009 |
| NPHS1 | 49 | 0.27 | 0.08 | 0.89 | 0.03 |

All variables with P value of ≤0.10, indicated in bold font, from the univariable analysis were included in one multivariable Cox model. The final multivariable Cox model was adjusted for the presence of anti-HLA antibodies, HLA-A,-B,-DR,-DQ antigen mismatches, repeat transplantation, deceased donation, recipient sex, recipient and donor age and induction therapy.

Abbreviation: see ***Table S1***.

***Figure S1.*** **Distribution of the A) measured raw MFI values and B) ratios above the cut-off per non-HLA antigen in all pretransplant sera (N=874).**


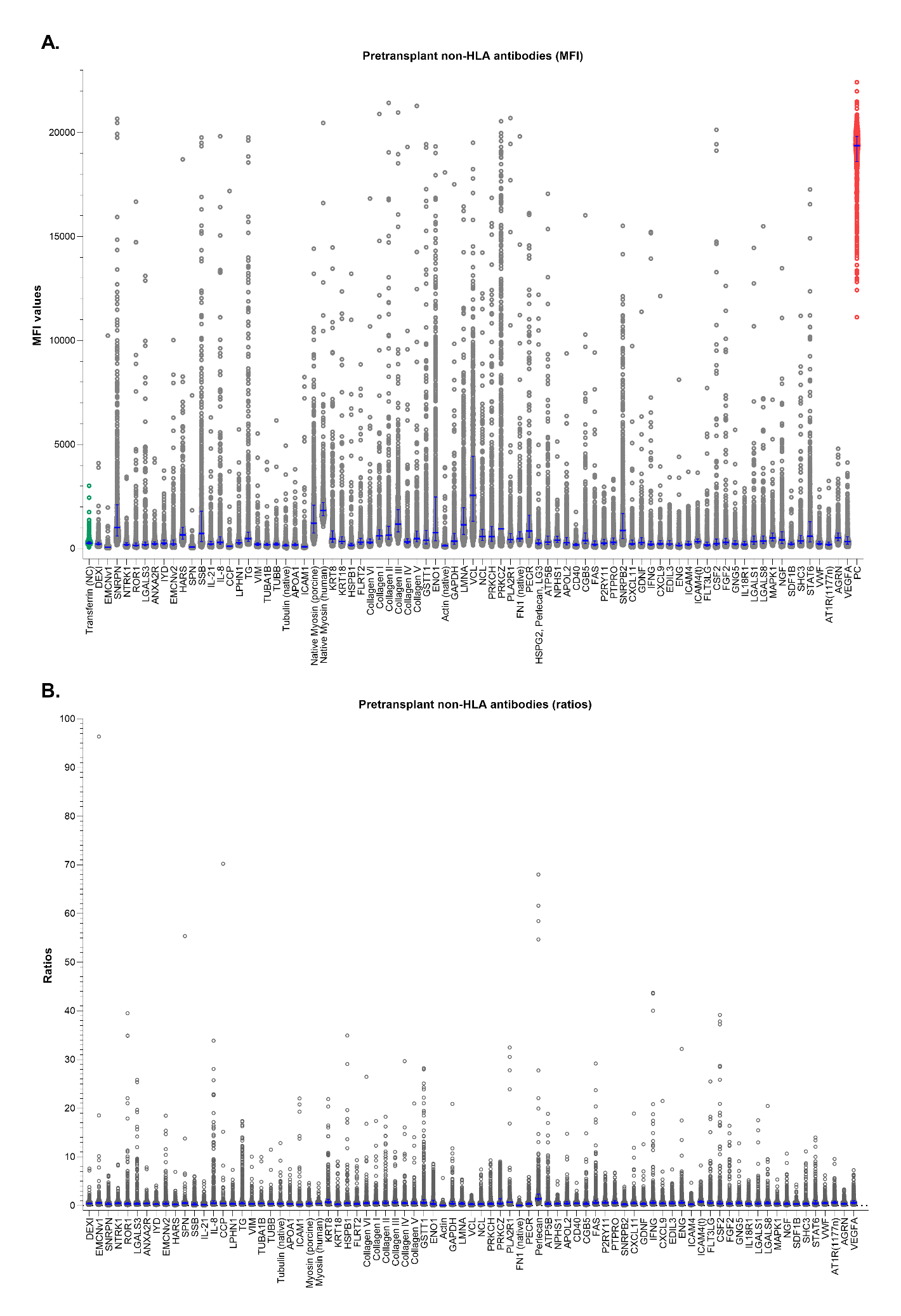


***Figure S2.*** **Pearson correlation matrix between the positive ratios of the antibodies against 81 non-HLA antigens and HLA-DSA.**

The graph displays only the correlations with a significant p-value after the Bonferroni correction. The circle's size and the intensity of the color represent the strength of the correlation between the different antibodies. The ratio for HLA-DSA per patient was calculated by summing the MFI of all DSAs divided by the MFI threshold of 500.


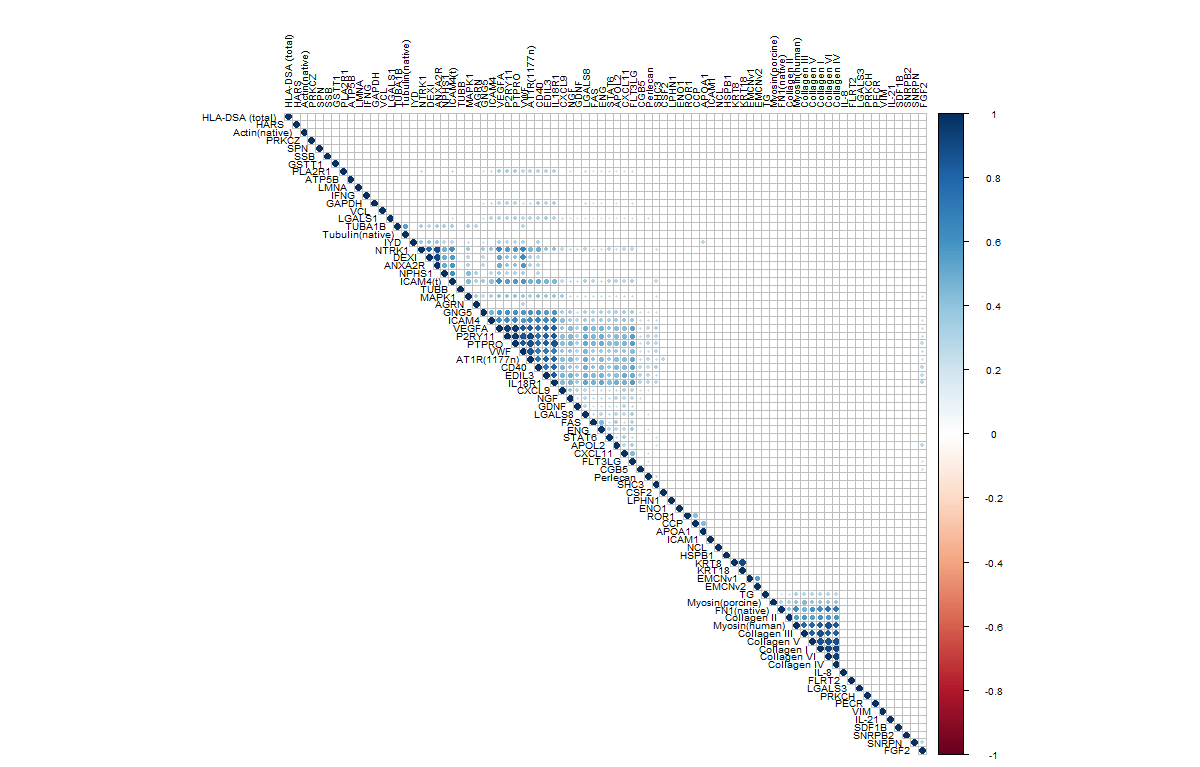


***Figure S3.* Comparison of the raw MFIs values for all antigen targets included in the kit at different time points.**


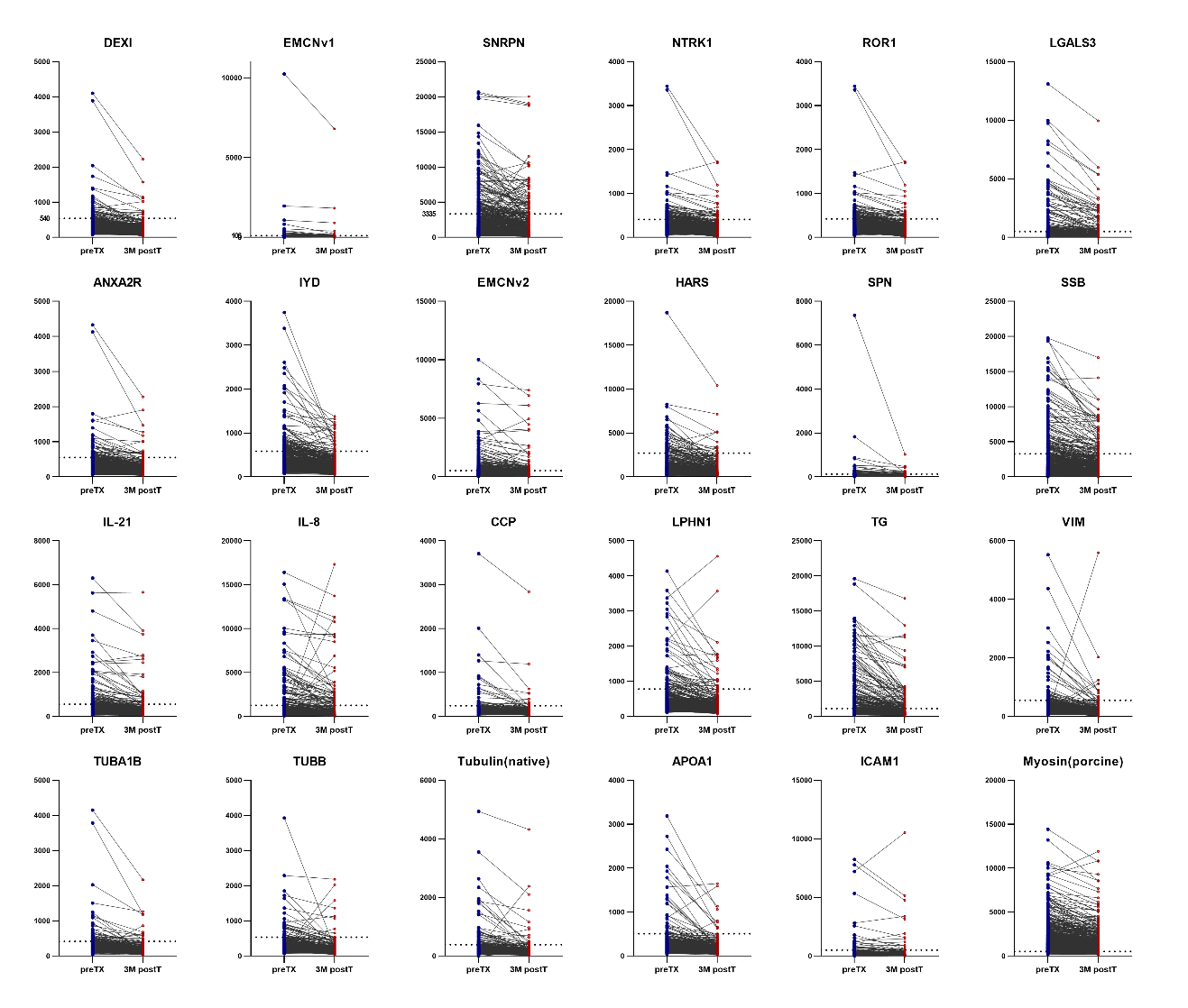

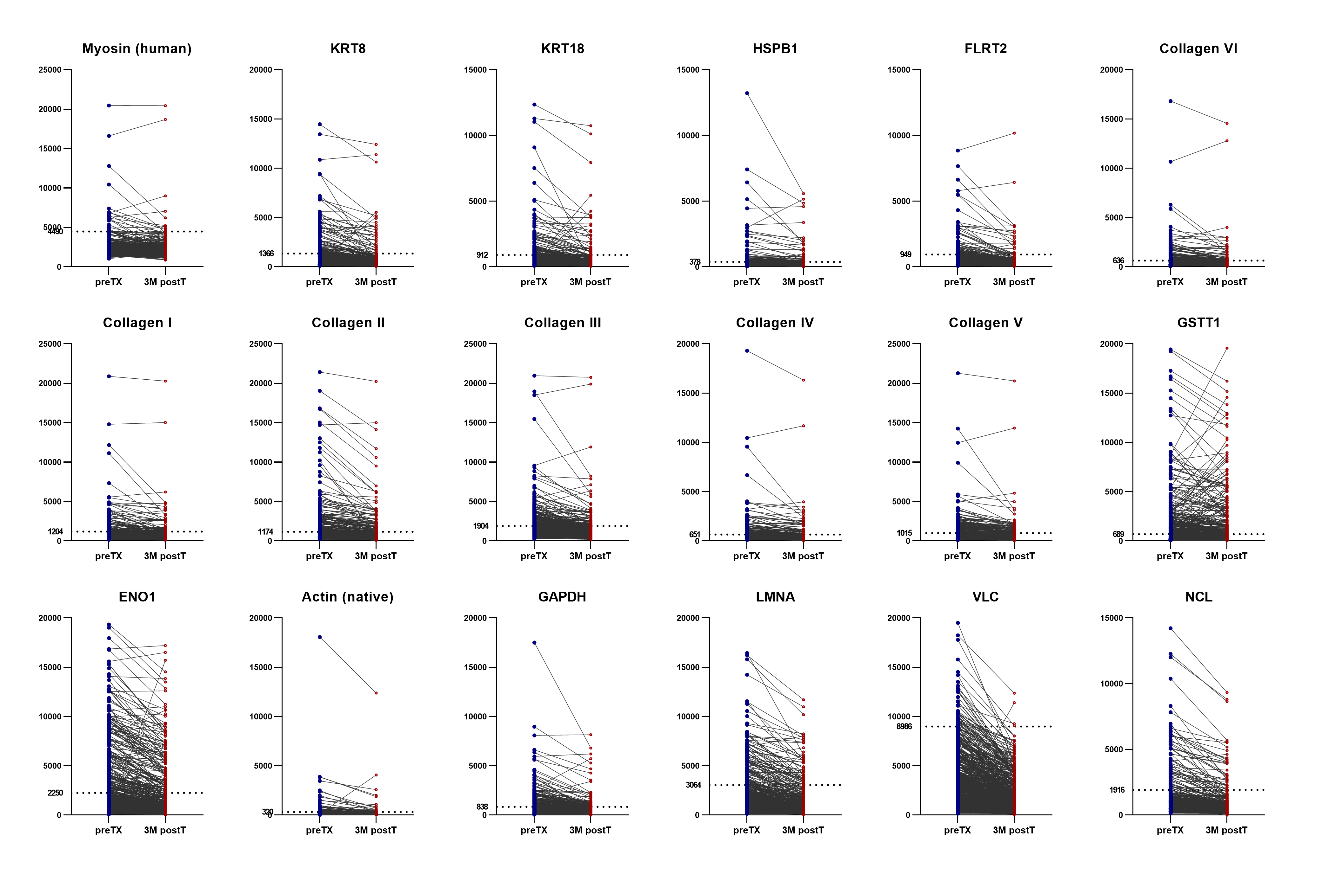
A. Paired t-test between pretransplant and 3-month posttransplant serum sample for each bead.

**
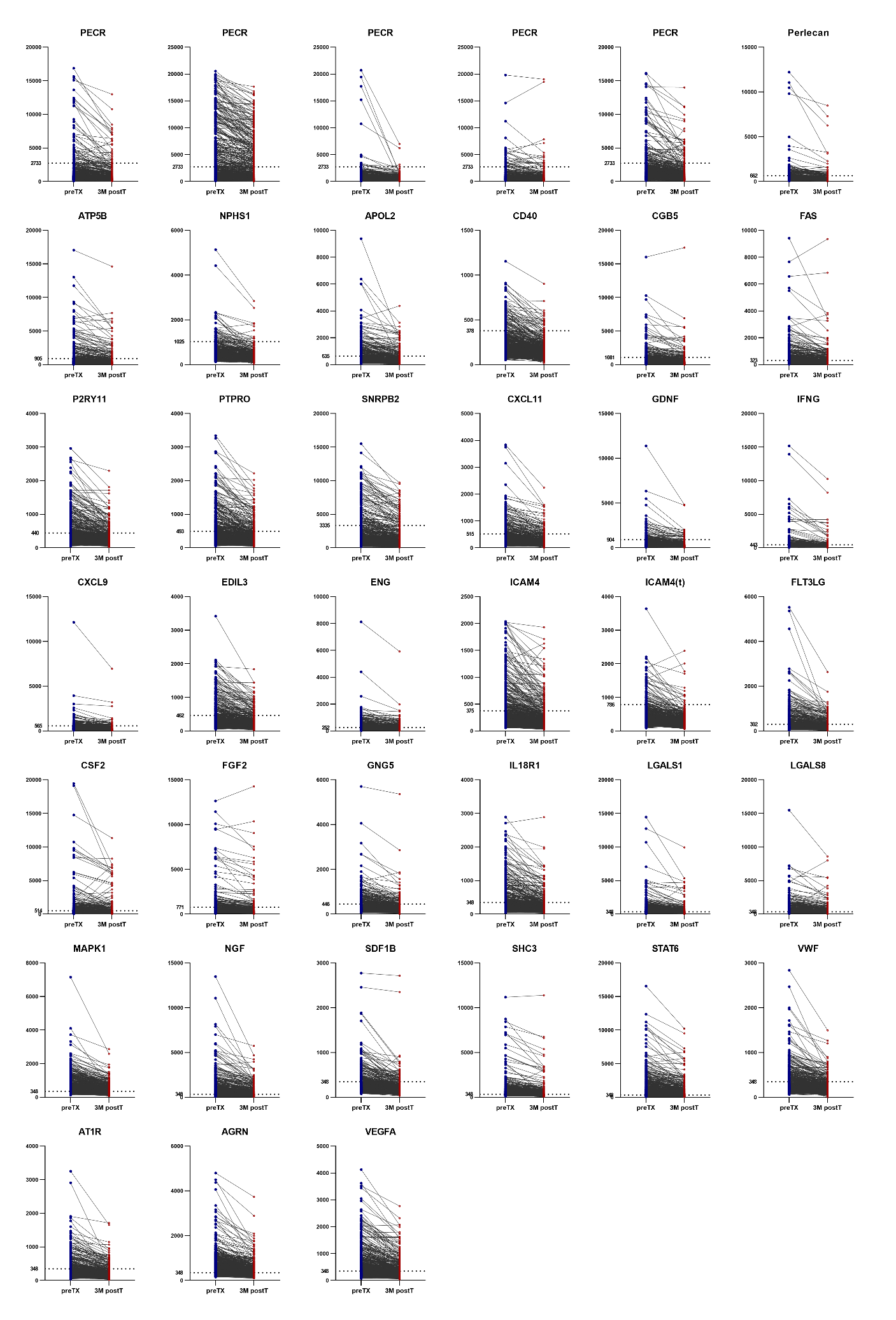
**

B. Paired t-test between 3-month and 1-year posttransplant serum sample for each bead.

**
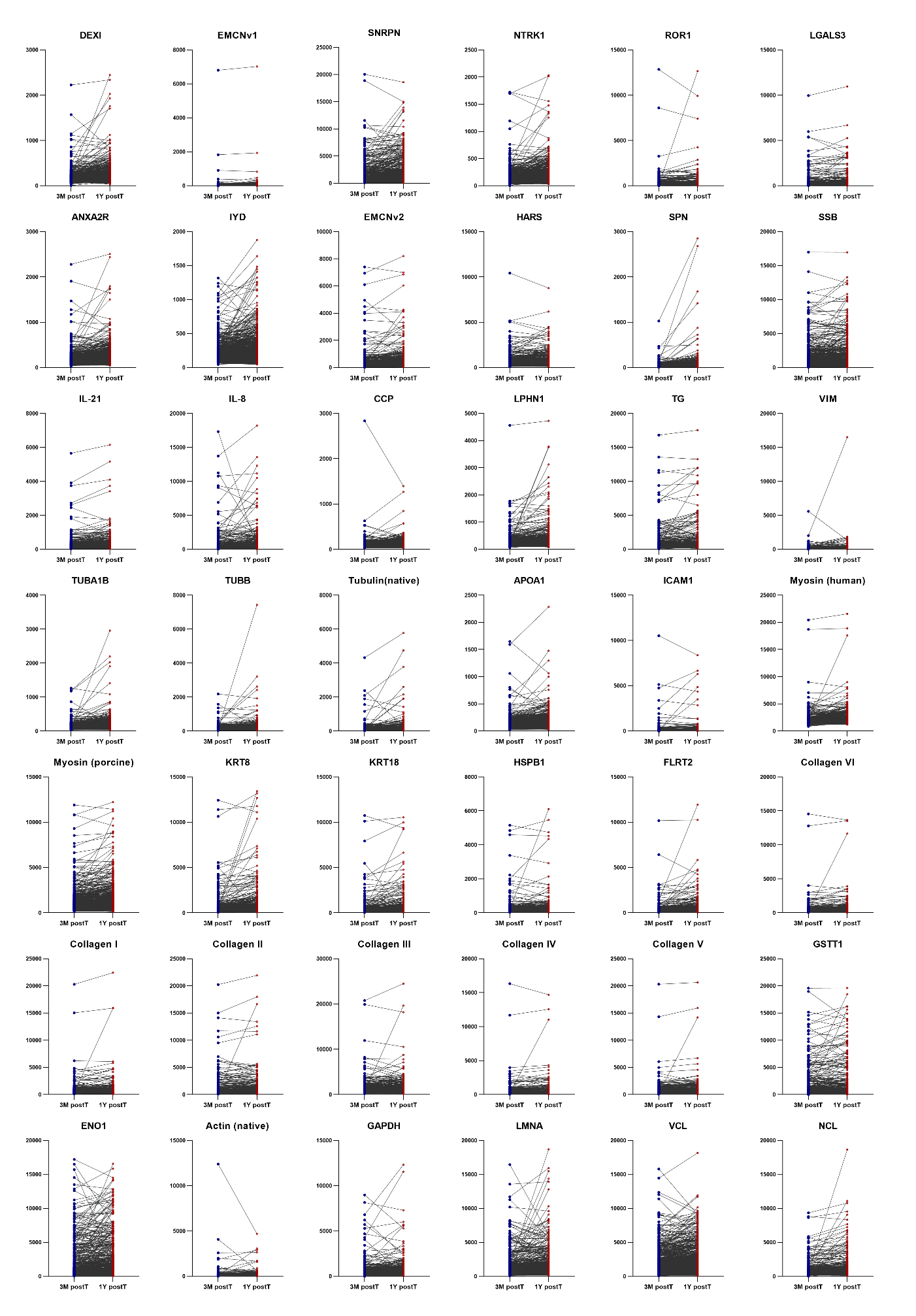
**


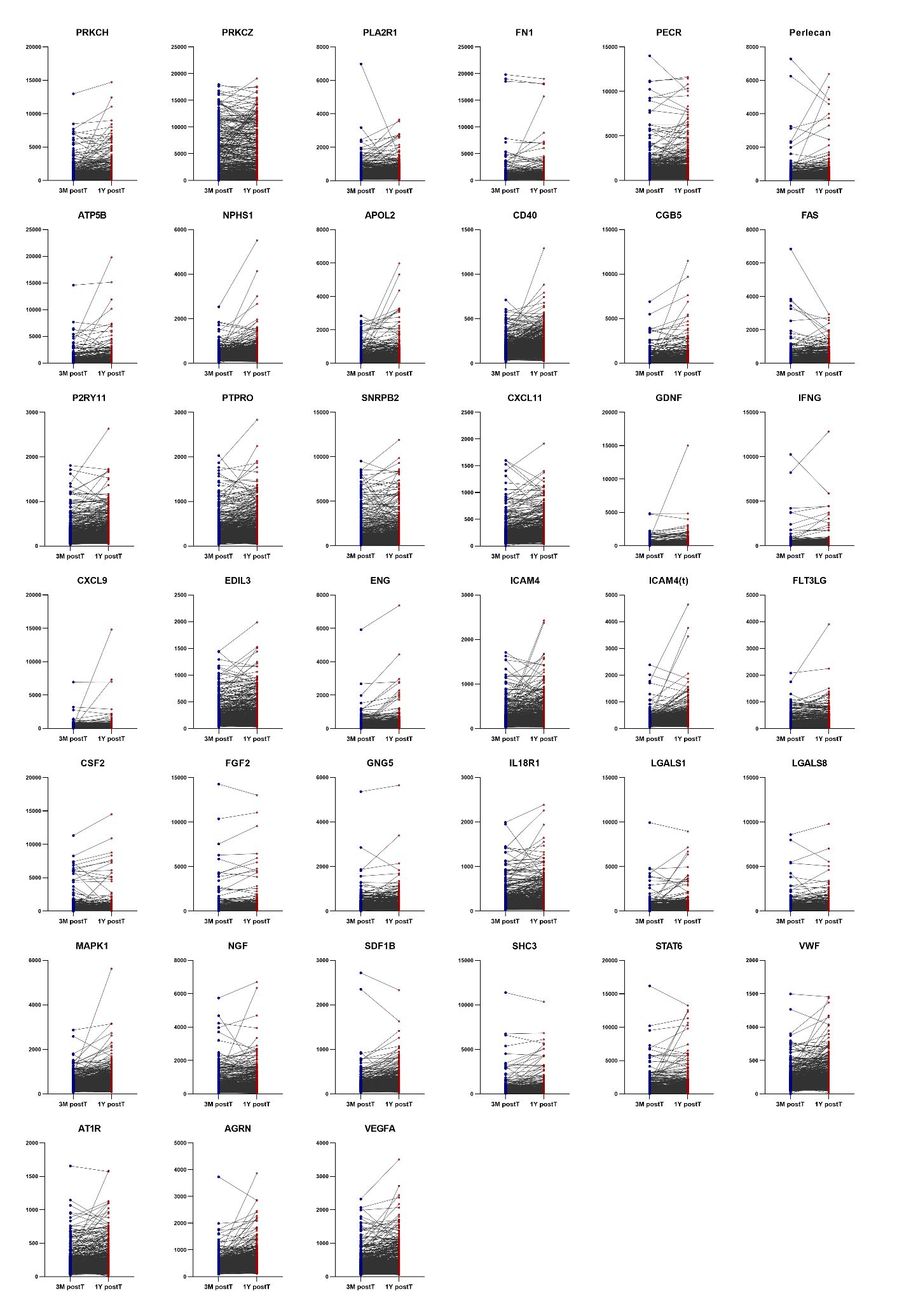

Supplement: Supplementary file 1 [file DataSheet_1.docx]
